# Signal profiling of the β_1_AR reveals coupling to novel signalling pathways and distinct phenotypic responses mediated by β_1_AR and β_2_AR

**DOI:** 10.1038/s41598-020-65636-3

**Published:** 2020-05-29

**Authors:** Viktoriya Lukasheva, Dominic Devost, Christian Le Gouill, Yoon Namkung, Ryan D. Martin, Jean-Michel Longpré, Mohammad Amraei, Yuji Shinjo, Mireille Hogue, Monique Lagacé, Billy Breton, Junken Aoki, Jason C. Tanny, Stéphane A. Laporte, Graciela Pineyro, Asuka Inoue, Michel Bouvier, Terence E. Hébert

**Affiliations:** 1 Department of Biochemistry and Molecular Medicine, Institute for Research in Immunology and Cancer (IRIC), Université de Montréal, Montréal, Québec Canada; 2 Department of Pharmacology and Therapeutics, McGill University, Montréal, Québec Canada; 3 Department of Medicine, Research Institute of the McGill University Health Centre, McGill University, Montréal, Québec Canada; 4 Institut de Pharmacologie and Department of Pharmacology-Physiology, Faculty of Medicine and Health Sciences, Université de Sherbrooke, Sherbrooke, Canada; 5 Department of Pharmacology and Physiology, Université de Montréal, Centre de Recherche de l’Hôpital Ste-Justine, Montréal, Canada; 6 Graduate School of Pharmaceutical Sciences, Tohoku University, Sendai, Japan

**Keywords:** Drug discovery, Pharmacology, Receptor pharmacology

## Abstract

A comprehensive understanding of signalling downstream of GPCRs requires a broad approach to capture novel signalling modalities in addition to established pathways. Here, using an array of sixteen validated BRET-based biosensors, we analyzed the ability of seven different β-adrenergic ligands to engage five distinct signalling pathways downstream of the β_1_-adrenergic receptor (β_1_AR). In addition to generating signalling signatures and capturing functional selectivity for the different ligands toward these pathways, we also revealed coupling to signalling pathways that have not previously been ascribed to the βAR. These include coupling to G_z_ and G_12_ pathways. The signalling cascade linking the β_1_AR to calcium mobilization was also characterized using a combination of BRET-based biosensors and CRISPR-engineered HEK 293 cells lacking the Gαs subunit or with pharmacological or genetically engineered pathway inhibitors. We show that both G_s_ and G_12_ are required for the full calcium response. Our work highlights the power of combining signal profiling with genome editing approaches to capture the full complement of GPCR signalling activities in a given cell type and to probe their underlying mechanisms.

## Introduction

Recent studies have established that GPCRs engage multiple signalling pathways and that ligands can discriminate between these pathways, which is now defined as functional selectivity or biased agonism^1–4^. Such effects have been characterized by examining the production of individual second messengers or more recently direct examination of proximal effector activation. Identification of signalling pathways beyond canonical effector systems has demonstrated the need to cast a broader net in studying signalling outcomes downstream of a given receptor. One such example is the recent recognition that, in addition to cAMP production, β-arrestin recruitment and ERK1/2 activation, the β_2_-adrenergic receptor (β_2_AR) can also promote calcium mobilization in a ligand-selective manner^5,6^. However, relatively little is known about functional selectivity for the β_1_AR. Although several papers have described functional selectivity for β_1_AR ligands^7,8^, a more comprehensive analysis of distinct pathways operating in living cells and the elaboration of a signalling signature is still lacking. Certain βAR antagonists, likely acting on cardiac β_1_AR, can activate a cardioprotective pathway involving β-arrestin signalling. Notably, alprenolol and carvedilol both acted as agonists for this pathway in a manner similar to clinically used agonists such as dobutamine and isoproterenol^9,10^. Thus carvedilol and alprenolol may be agonists for β_1_AR signalling pathways which might underlie their cardioprotective mechanisms in heart failure, in contrast to numerous other βAR antagonists without such pleiotropic activity. Carvedilol is known to activate ERK1/2 via a pathway involving β-arrestin^11^. These observations argue that such functional selectively is worth exploring further for both receptors which may have clinical impact in a number of diseases.

Historically, most GPCRs have been defined as being coupled to a primary heterotrimer G protein partner^12^. However, it is clear that such definitions are oversimplifications of the actual wiring of receptors into multiple signalling pathways^13^. Therefore, to generate a more global understanding of the repertoire of signalling pathways that can be engaged by a given receptor, and of the possible implications of signalling diversity generated by different ligands, a broader approach taking into account multiple GPCR effectors and downstream signalling cascades is needed^14–17^. Toward that end, we present here an integrated platform of BRET-based biosensors detecting both proximal and distal outputs downstream of the β_1_AR. We combine this with CRISPR/Cas9 genome editing^18–22^ and genetically engineered dominant negative approaches to target G proteins to examine the functional consequences of removing individual G proteins on signalling outcomes.

BRET-based biosensors have become a convenient and robust method for characterizing both canonical and novel signalling pathways modulated by GPCRs^15–17,23,24^. These biosensors have distinct designs that allow detection of intermolecular interactions between proteins, intramolecular structural reorganization resulting from protein partners, and ligand or second messenger binding. We show that the combination of such biosensors, including two specifically developed for the present study, with genome editing approaches facilitates detection of new signalling pathways downstream of a given receptor as well as their functional consequences.

## Results

### General approach

The purpose of the study was to obtain a broad profiling of the signalling activity of the β1-adrenergic receptor to generate a more comprehensive map of its signalling potential. We chose the β_1_AR as an example of an extensively studied GPCR to explore the possibility that, in addition to well characterized signalling pathways which have been studied for many decades (i.e.: G_s_/cAMP and β-arrestin), new pathways might also be revealed (Supplementary Fig. S1). To explore the potential pleiotropic receptor signalling, seven β-adrenergic ligands were tested across a panel of identified signalling cascades. For this purpose, we used a variety of BRET-based sensors that allowed us to monitor 10 different G protein subtypes (including at least one from each of the 4 Gα subunit classes), β-arrestin2, and the activity of 4 effectors downstream of engaged G proteins. To probe the functional significance of the newly identified β-adrenergic-activated signalling pathways, we combined the use of the biosensors with a CRISPR-Cas9-generated cell line lacking Gα_s_, the cognate G protein subtypes engaged by β_1_AR as well as a novel engineered dominant negative construct selectively inhibiting Gα_12/13_.

### G protein pathways engaged by the β_1_AR

**Figure 1:**
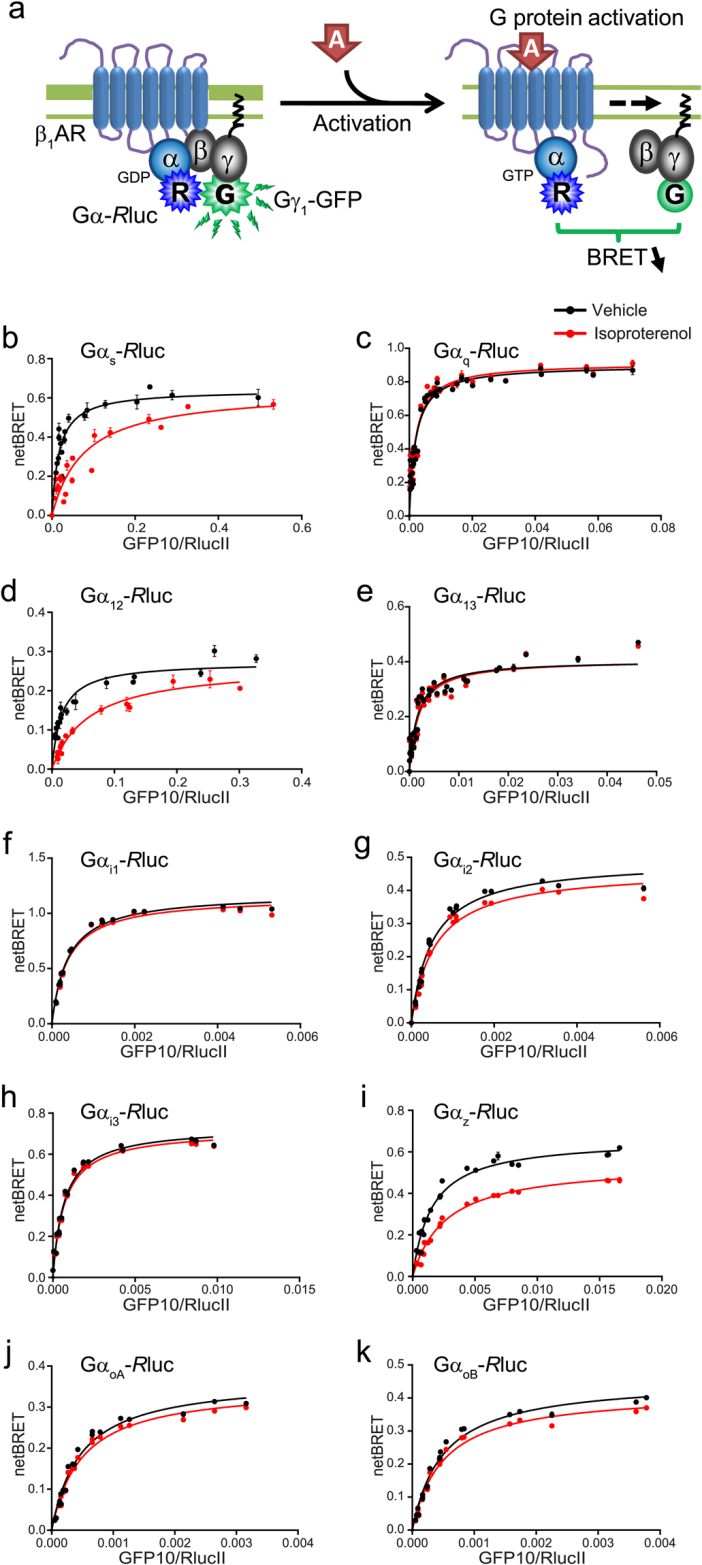
*Identification of the Gα protein involved in β*_1_*AR signalling*. **(a)** Schematic representation of the Gα-Rluc/Gγ-GFP biosensor used to identify the Gα proteins involved in β_1_AR signalling. (**b–k**) HA-β_1_AR HEK 293 stable cell lines were transfected with a constant amount of Gα-*R*luc (BRET donor) and untagged Gβ_1_, along with increasing amounts of Gγ_1_-GFP construct (BRET acceptor). Cells were stimulated (red curves) or not (black curves) with 1 μM isoproterenol and BRET values collected. NetBRET values were calculated by subtracting the background BRET signal detected in cells expressing the *R*luc-fused constructs alone (donor-*R*luc) from the BRET values obtained in cells expressing the energy donor and acceptor (donor-*R*luc and acceptor-GFP). BRET titration curves were generated for 10 different Gα subunits: Gα_s_ (**b**), Gα_q_ (**c**), Gα_12_ (**d**), Gα_13_ (**e**), Gα_i1_ (**f**), Gα_i2_ (**g**), Gα_i3_ (**h**), Gα_z_ (**i**), Gα_oA_ (**j**) and Gα_oB_ (**k**). Values represent mean ± SEM of 3 independent experiments performed in triplicate. Responses for Gα_s_, Gα_i2_, Gα_z_ and Gα_12_ were further analyzed in subsequent sections.

In order to assess which G protein subtypes were activated by the β_1_AR, we used a panel of biosensors designed to capture the activation state of the heterotrimer by measuring the physical separation of Gα and Gβγ subunits via BRET^25^. Figure 1 shows BRET titration curves for 10 different Gα subunits, indicating that the βAR agonist isoproterenol leads to a robust activation of G_s_, G_12_ and G_z_, as measured by a decrease in BRET. Smaller decreases in BRET in response to isoproterenol were also observed for other G proteins (G_i2_, G_oA_ and G_oB_). No responses were observed for G_q_, G_13_, G_i1_ or G_i3_. The lack of activation of different G protein isoforms did not result from insufficient biosensor sensitivity since robust responses were detected for the D2 dopamine receptor toward all Gα_i_ subtypes (Supplementary Fig. S2), for TPα receptors toward both G_12_ and G_13_ (Supplementary Fig. S3a,c) as well as for other GPCRs toward G_q_^17^, G_13_^26^ and G_i_^17,27^. To further characterize the activation of these G proteins and their downstream effector pathways by a panel of βAR ligands, full concentration-response curves and kinetic measurements were obtained using both the G protein sensors described above and biosensors detecting downstream events representative for each of these pathways.

### G_s_ signalling

**Figure 2:**
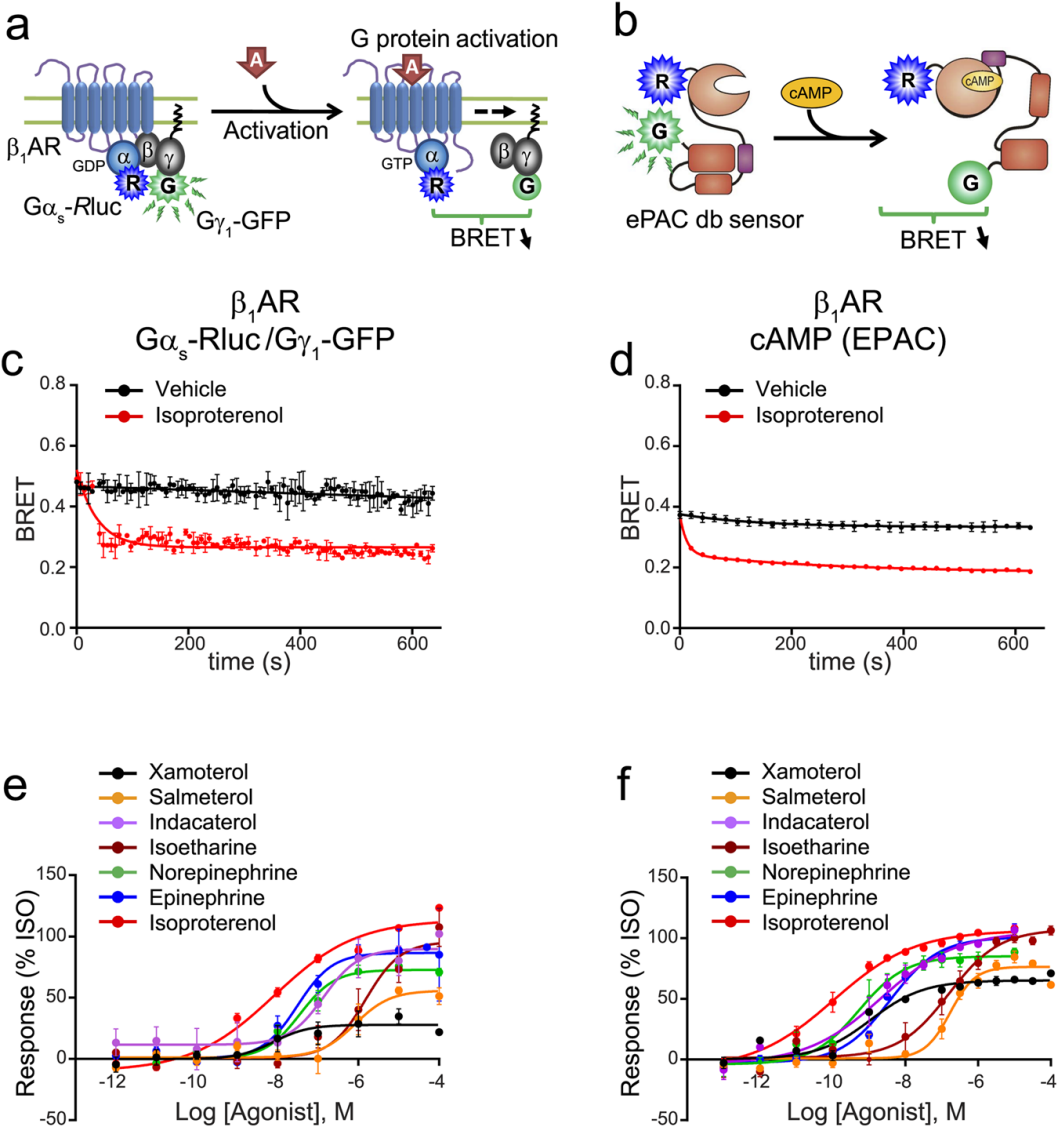
*Gα*_*s*_ *activation and cAMP production induced by the β*_*1*_*AR*. (**a**) Schematic representation of the Gα_s_-*R*luc/Gγ_1_-GFP biosensor used to study the Gα_s_ induced β_1_AR signalling. (**b**) Schematic representation of the cAMP biosensor used to study the cAMP increase induced by the β_1_AR Gα_s_ activation. HEK 293 cells were transfected with (**c**,**e**) Gα_s_-*R*luc, Gγ_1_-GFP and untagged Gβ_1_ or with the (**d**,**f**) EPAC biosensor, along with the β_1_AR. Kinetic curves represent time course of (**c**) Gα_s_ activation (vehicle and isoproterenol, n=3) or (**d**) cAMP accumulation (vehicle and isoproterenol, n=3) expressed as absolute BRET ratio. Concentration-responses curves were generated for (**e**) Gα_s_ activation and (**f**) cAMP accumulation following β_1_AR activation by the indicated ligands. Data were normalized to maximal isoproterenol response, which was taken as 100%, and are expressed as mean ± SEM values. Detail of the number of experiments, maximal responses, pEC_50_ values and statistical comparisons of curve parameters for different ligands are provided in Supplementary Tables S1 and S2.

To probe the G_s_ signalling pathway, an EPAC biosensor sensitive to cAMP levels^28,29^ was used in conjunction with the G_s_ activation biosensor (Fig. 2a,b). Similar activation kinetics were detected using both biosensors (Fig. 2c,d) and the effects of both full and partial agonists were revealed by the concentration-responses curves (Fig. 2e,f). A general pattern of concordance for both biosensors (Supplementary Tables S1 and S2) was observed with respect to efficacy and potency, although the differences between full and partial agonists were reduced when using the EPAC biosensor, consistent with the notion of signal amplification at the level of cAMP production.

### G_i_ signalling

**Figure 3:**
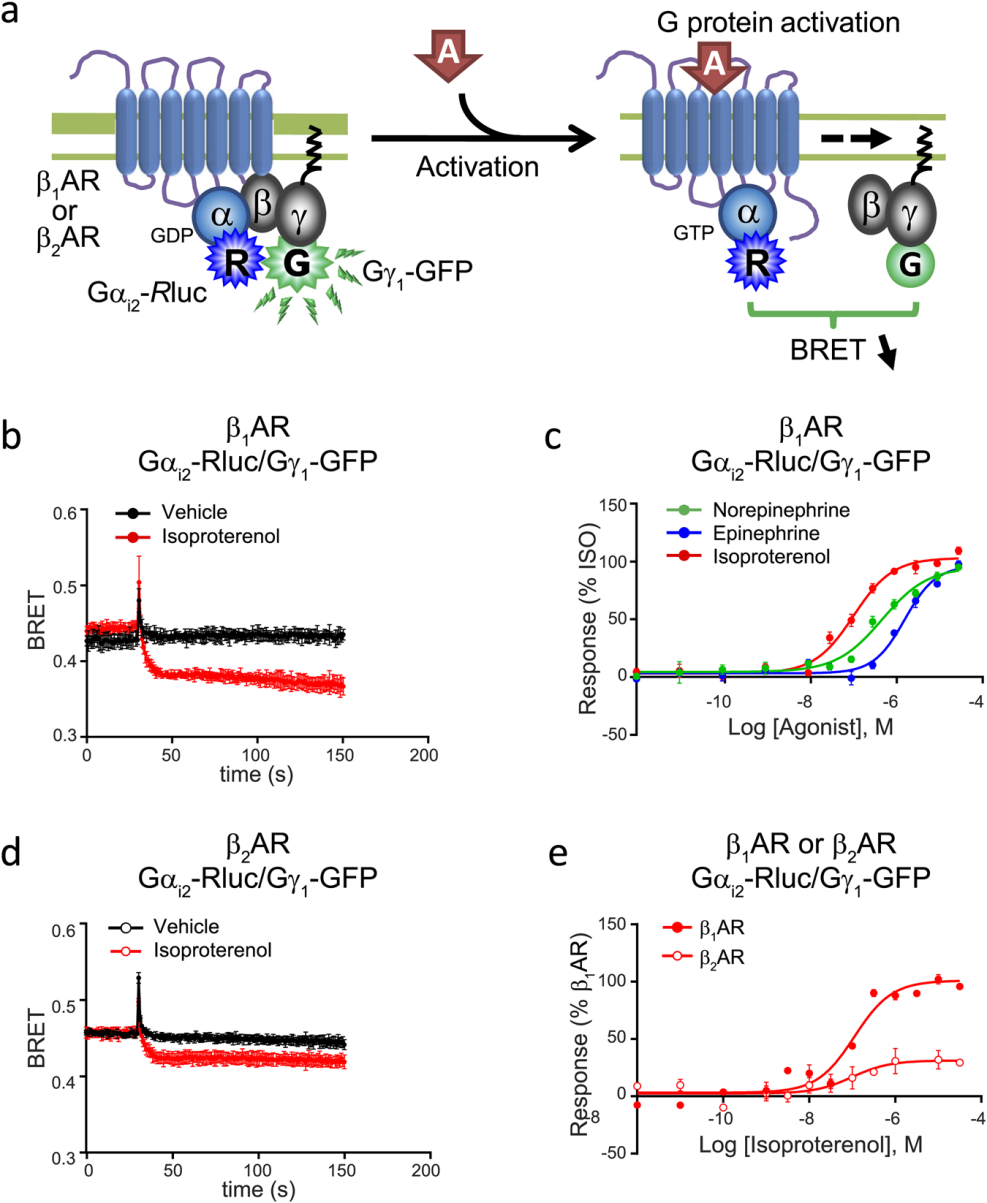
*Gα*_*i*2_*-induced activation by the β*_1_*AR and β*_2_*AR*. (**a**) Schematic representation of the Gα_i2_-Rluc/Gγ_1_-GFP biosensor used to study the Gα_i_ induced β_1_AR and β_2_AR signalling. HEK 293 cells were transfected with (**b-e**) Gα_i2_-*R*luc, Gγ_1_-GFP and untagged Gβ_1_, along with (**b-c,e**) β_1_AR or (**d-e**) β_2_AR. (**b,d**) Kinetics curves represent time course of Gα_i2_ activation by (**b**) β_1_AR (vehicle and isoproterenol, n=3) or (**d**) β_2_AR (vehicle and isoproterenol, n=3) expressed as absolute BRET ratios. (**c**) Concentration-responses curves for Gα_i2_ activation following β_1_AR activation by indicated ligands. (**e**) Concentration-responses curves for Gα_i2_ activation following isoproterenol-induced activation of β_1_AR or β_2_AR (n=2). Data were normalized to maximal isoproterenol response, which was take as 100%, and are expressed as mean ± SEM values. Detail (**c**) of the number of experiments, maximal responses, pEC_50_ values and statistical comparisons of curve parameters for different ligands are provided in Supplementary Tables S1 and S2.

For each of the canonical PTX-sensitive Gi family members (G_i1–3_, G_oA_ and G_oB_, Fig. 3a), concentration-response curves were generated to further characterize the weak activation detected for some of these isoforms in BRET titration experiments. Reproducible kinetic responses and concentration/response curves could only be obtained for G_i2_ (Fig. 3b,c, Supplementary Tables S1, S2). Interestingly, only three agonists (isoproterenol, epinephrine and norepinephrine) could elicit responses from G_i2_, suggesting more efficient coupling to G_s_. Of note, isoetharine and indacaterol, which were close to full agonist on G_s_ activation and cAMP production could not evoke any G_i_ activation. The fact that we only detected G_i2_ is not because this biosensor is more sensitive than those for other G_i_ family members, as we could detect a more robust response for G_i1_ than G_i2_ for the D2-dopamine receptor which can activate all G_i_ subfamily members (Supplementary Fig. S2). Although not previously reported for the β_1_AR, previous studies have also demonstrated coupling of the β_2_AR to G_i_ using conventional assays^30,31^ and we could also detect β_2_AR coupling to G_i2_ using the BRET-based sensor (Fig. 3d,e).

### G_z_ signalling

**Figure 4:**
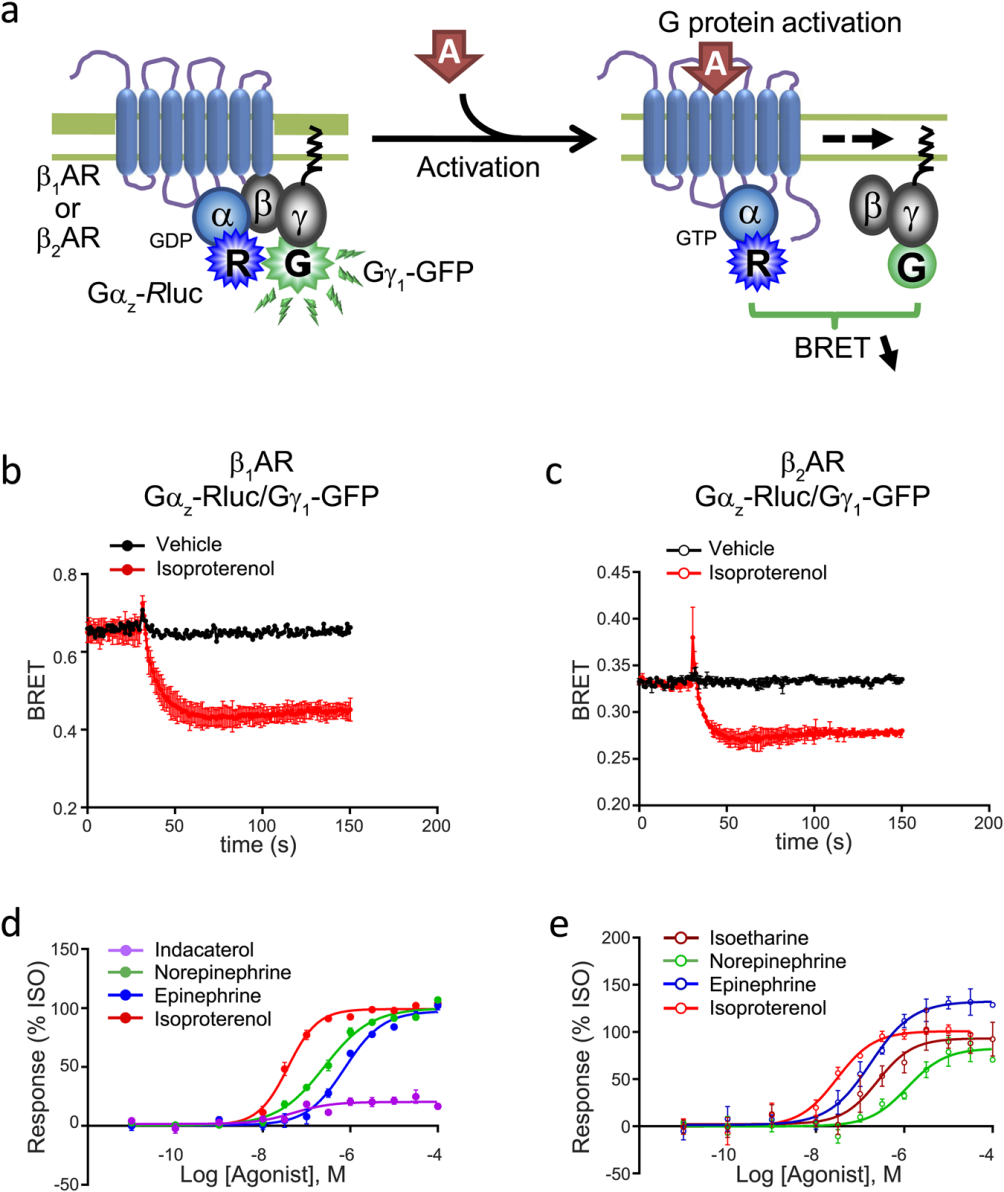
*Gα*_*z*_*-induced activation by the β*_*1*_*AR and β*_*2*_*AR*. (**a**) Schematic representation of the Gα_z_-Rluc/Gγ_1_-GFP biosensor used to study the Gα_z_ induced βAR signalling. HEK 293 cells were transfected with Gα_z_-*R*luc, Gγ_1_-GFP and untagged Gβ_1_, along with (**b,d**) β_1_AR or (**c,e**) β_2_AR. Kinetic curves represent time course of Gα_z_ activation by (**b**) β_1_AR (vehicle n = 1; isoproterenol n = 2) or (**c**) β_2_AR (vehicle and isoproterenol, n = 2) expressed as absolute BRET ratios. Concentration-responses curves for Gα_z_ activation following (**d**) β_1_AR or (**e**) β_2_AR activation by indicated ligands. Data were normalized to maximal isoproterenol response, which was take as 100%, and are expressed as mean ± SEM values. Details of the number of experiments, maximal responses, pEC_50_ values and statistical comparisons of curve parameters for (**d**) β_1_AR activation by different ligands are provided in Supplementary Tables S1 and S2. For (**e**) β_2_AR activation, n = 3 for all ligands.

G_z_ is an atypical member of the G_i_ family as it is insensitive to pertussis toxin. We next investigated if this G protein could be activated by the β_1_AR. Our BRET-based biosensor (Fig. 4a) detected robust time- and concentration-dependent activation of G_z_ by the β_1_AR (Fig. 4b,d). The concentration-response curves in Fig. 4d show that, in addition to the three full agonists for G_s_, indacaterol also acted as a weak partial agonist for G_z_. The lack of detection of the effects of other partial agonists was not caused by differential dynamic windows in each assay as isoproterenol responses were similar in both the G_s_ and G_z_ sensors (compare Fig. 1b with Fig. 1i). As G_z_ coupling has never previously been documented for any βAR subtype, we also examined the ability of the β_2_AR to engage G_z_. The kinetics of activation in response to isoproterenol were similar for both receptor subtypes (Fig. 4b,c). Further, as expected, the rank order of potency of the ligands was distinct for each receptor subtype, generally reflecting their relative affinities for the two receptors (Fig. 4d,e). However, one notable exception was that despite its characterization as a β_2_AR-selective ligand, indacaterol^32,33^ only produced a response in the β_1_AR, indicating that selectivity also depends on the signalling pathway examined; a further manifestation of functional selectivity. A similar effect was observed for isoetharine, activating G_s_ through both β_1_- and β_2_AR (albeit with lower potency for the β_1_AR), but only resulting in G_z_ coupling for the β_2_AR (Fig. 4e).

### G_12_ signalling

**Figure 5:**
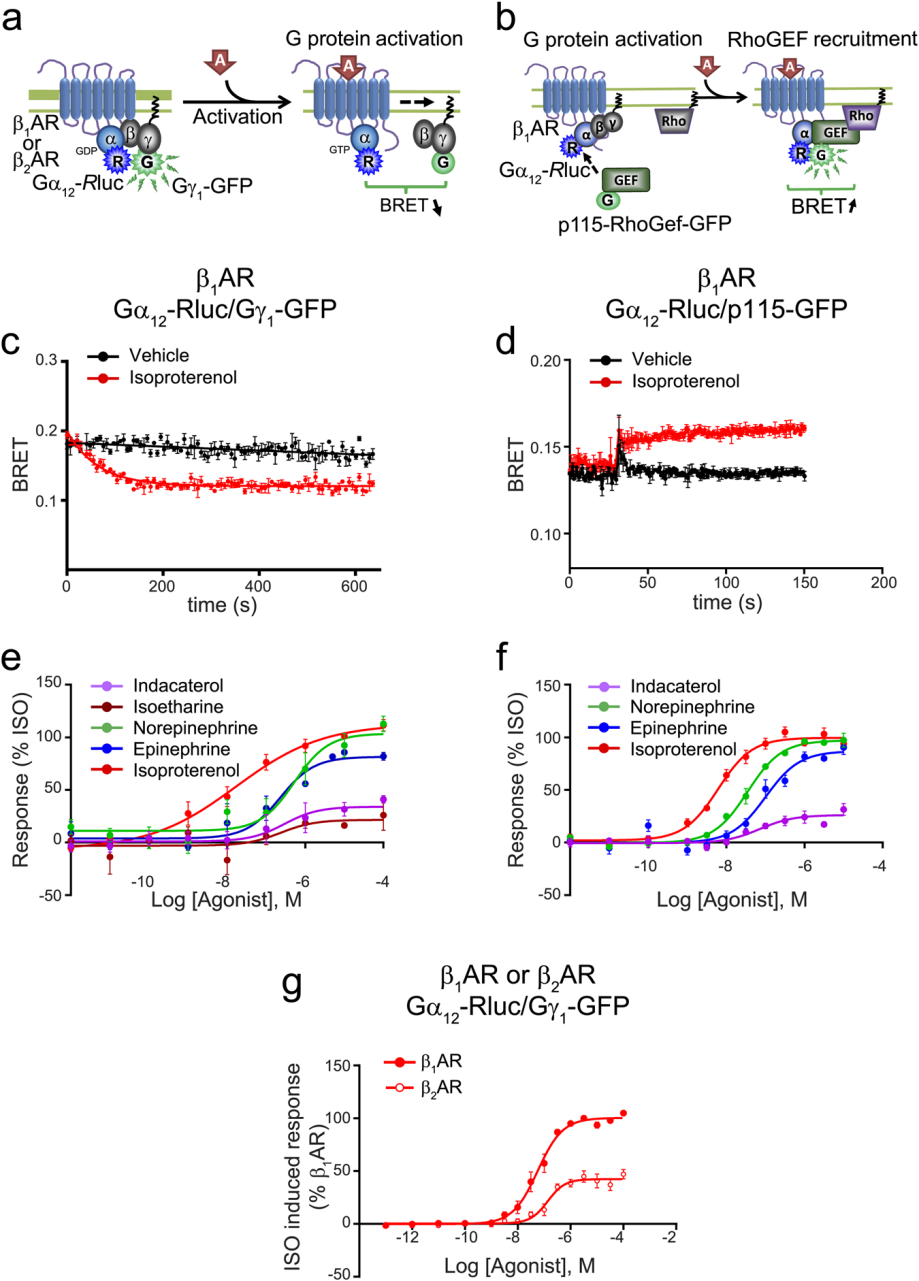
*Gα*_12_*-induced activation by the β*_1_*AR*. (**a**) Schematic representation of the Gα_12_-*R*luc/Gγ_1_-GFP biosensor used to study the Gα_12_ induced βAR signalling. (**b**) Schematic representation of the Gα_12_-Rluc/p115-RhoGef-GFP (p115-GFP) biosensor used to study the Gα_12_ induced βAR signalling. HEK 293 cells were transfected with (**c,e,g**) Gα_12_-*R*luc, Gγ_1_-GFP and untagged Gβ_1_ or with (**d,f**) Gα_12_-Rluc, p115-GFP and untagged Gγ_1_ and Gβ_1_, along with β_1_AR or (**g**) β_2_AR. Kinetic curves represent time course of (**c**) Gα_12_ activation (vehicle and isoproterenol n = 3) or (**d**) Gα_12_-p115 biosensor activation (vehicle n = 2, isoproterenol n = 3), expressed as absolute BRET ratio. Concentration-responses curves for (**e**) Gα_12_ activation or (**f**) Gα_12_-p115 biosensor activation following β_1_AR activation by indicated ligands. (**g**) Concentration-responses curves for Gα_12_ activation following isoproterenol-induced β_1_AR or β_2_AR stimulation (n = 6). Data were normalized to maximal isoproterenol response (100%), and are expressed as mean ± SEM values. Detail (**e,f**) of the number of experiments, maximal responses, pEC_50_ values and statistical comparisons of curve parameters for (**e,f**) β_1_AR activation by different ligands are provided in Supplementary Tables S1 and S2.

Another novel feature of β_1_AR signalling captured by our biosensor panel was the activation of G_12_ (Fig. 5a,c,e,g) with no detectable activation of G_13_ (compare Fig. 1d,e). A previous report^34^ showed a similar ability of GPR35 to discriminate between G_12_ and G_13_. The lack of activation of G_13_ by the β_1_AR did not result from lower sensitivity of the biosensor as we could readily detect its activation by a known activator, the TPα receptor (TPαR, Supplementary Fig. S3a,c). To confirm and further characterize G_12_ activation, an additional assay monitoring downstream engagement of p115-RhoGEF was designed (Fig. 5b) and again validated using TPαR (Supplementary Fig. S3b,d). This biosensor monitors recruitment of the rgRGS domain of the G_12/13_ effector, p115-RhoGEF, directly to Gα_12_ by monitoring BRET between Gα_12_-Rluc and p115-RhoGEF-GFP10. The expression of the biosensor components did not affect cell surface receptor expression (Supplementary Fig. S4a,b). Rapid G_12_ activation kinetics and recruitment of p115-RhoGEF were noted in response to isoproterenol (Fig. 5d,f). The kinetics of the two biosensors were quite different. It is difficult to make direct kinetic comparisons between biosensors based on different designs. In the case of Fig. 5c, the G12 activation biosensor is based on dissociation of Gα from Gβγ whereas the biosensor for RhoGEF is based on recruitment of a subdomain of p115-RhoGEF to the Gα (Fig. 5d). The dynamics of such interactions are most likely different and possibly explain the difference in the kinetics observed. The other compounds tested displayed similar potencies and efficacies for either of the two G_12_ pathway biosensors, with the exception of the weak partial agonist isoetharine, where activity could only be detected for the G_12_/Gβγ sensor (compare Fig. 5e,f). Compounds had similar rank order of potencies and efficacies as those observed for G_z_.

**Figure 6:**
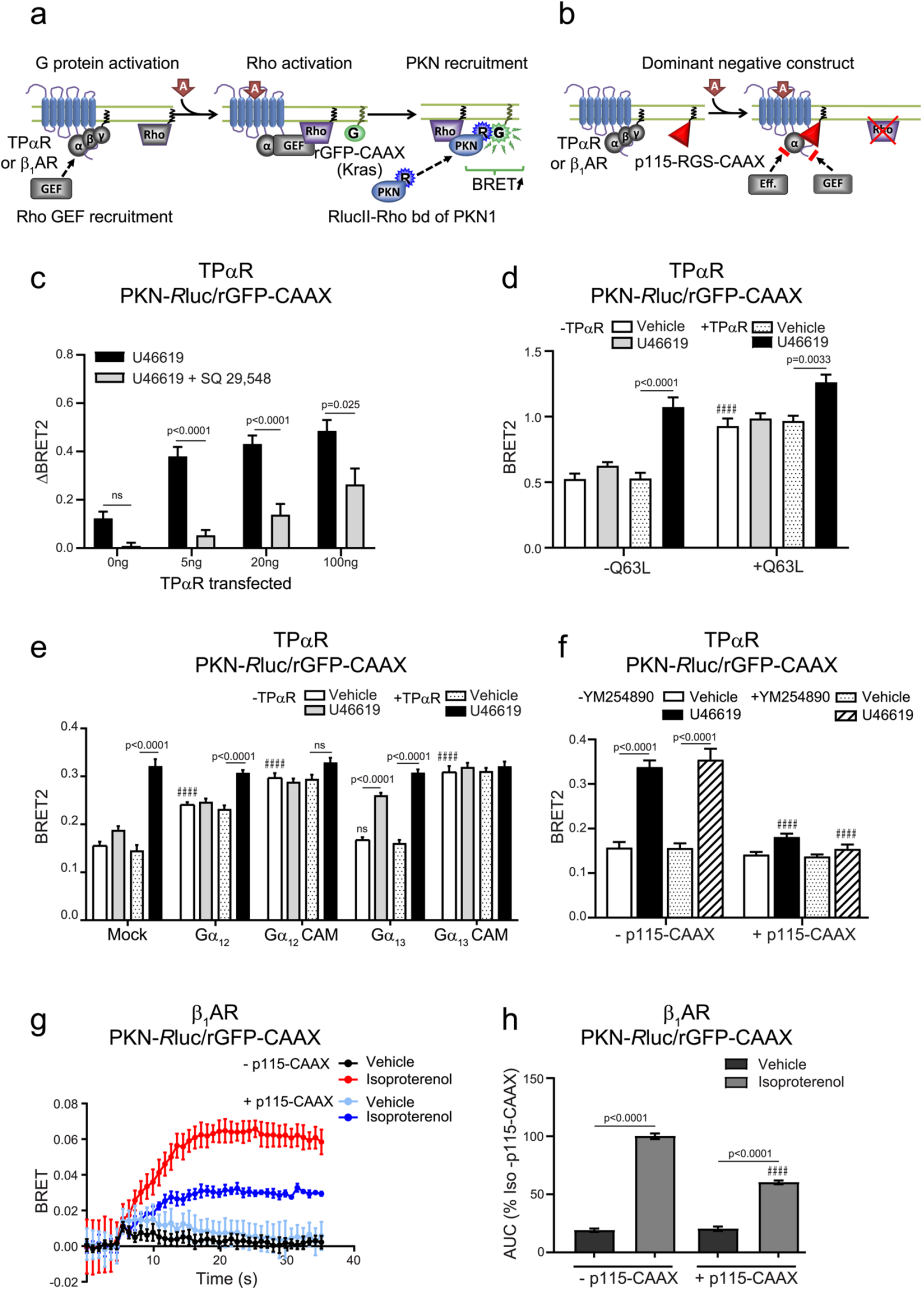
*PKN-recruitment based biosensor to monitor receptor-induced activation of Rho signalling for TPαR and β*_1_*AR*. (**a**) Schematic representation of the PKN-recruitment based biosensor. (**b**) Schematic representation of the mode of action of p115-RGS-CAAX construct (P115-CAAX) as a dominant negative construct for G protein signalling. HEK 293 cells were transfected with PKN-*R*lucII and rGFP-CAAX along with either (**c–f**) HA-TPαR or (**g,h**) β_1_AR, in the presence or absence of (**d**) constitutively active RhoA mutant (Q63L), (**e**) Gα_12_, Gα_13_ or their constitutively active (CAM) versions (Gα_12_CA Q231L and Gα_13_CA Q226L) or (**f-h**) p115-CAAX. **(c)** Treatment with the TP antagonist SQ 29,548 inhibits PKN recruitment following TPαR activation by U46619. 0 ng represents the activity of endogenously expressed receptor. Data are expressed as ΔBRET signal and are the mean ± SEM (n = 3). Statistical comparisons for antagonistic effect were done using two-way ANOVA followed by post-hoc comparison with Sidak’s test. **(d,e)** PKN recruitment to the plasma membrane upon U46619-induced TPαR activation in the presence or absence of (**d**) constitutively active RhoA mutant (Q63L) or (**e**) Gα_12_, Gα_13_ or their CAM versions. Data are expressed as BRET signal and are mean ± SEM ((**d**) n = 6, (**e**) n = 3). Statistical comparisons were done using two-way ANOVA followed by post-hoc comparison with Tukey’s test. #### p < 0.0001 compared to (**d**) Q63L or (**e**) TPαR Vehicle Mock. (**f**) Inhibition of PKN recruitment upon U46619-induced TPαR activation in the presence of the dominant negative p115-CAAX construct or the Gq inhibitor YM-254890. Data expression and statistical comparisons were done as in (**d,e**). n = 4, #### p < 0.0001 compared to -p115-CAAX. **(g)** Kinetics of PKN recruitment upon isoproterenol-induced β_1_AR activation in the presence of the dominant negative p115-CAAX (representative of n = 3). **(h)** β_1_AR-mediated PKN recruitment in the presence of p115-CAAX. Data are expressed as the area under the curve (AUC), calculated from 30 sec kinetics of 1 µM isoproterenol stimulation, and are the mean ± SEM (n = 4). #### p < 0.0001 compared to -p115-CAAX. ns: non-significant.

As for G_z_ coupling discussed above, engagement of G_12_ had never been previously described for any βAR subtype. Therefore, the ability of the β_2_AR to activate this pathway was also examined. The β_2_AR was also capable of activating G_12_ with potencies that were similar between the two receptors (Fig. 5g). To further characterize the downstream consequences of the engagement of Gα_12_ by the β_1_AR, we next assessed activation of the Rho pathway, a known downstream effector of G_12_. Recruitment of PKN to the plasma membrane was measured using an ebBRET^17^ biosensor based on PKN translocation to the plasma membrane upon Rho activation. This sensor monitors RlucII-tagged PKN (Rluc-PKN) density at the plasma membrane, which is labelled with a BRET acceptor, *Renilla reniformis* GFP (rGFP-CAAX, Fig. 6a). TPαR, a known activator of the G_12_/Rho/PKN pathway^35–37^, was used to validate this biosensor. As shown in Fig. 6c, agonist-mediated activation of TPαR led to an increased BRET signal, reflecting membrane recruitment of PKN. This response was blocked by the TPαR antagonist, SQ 29548. Consistent with the ability of this biosensor to detect Rho activity, a constitutively active mutant form of RhoA, Q63L-RhoA^38^, promoted robust BRET from the PKN-based sensor (Fig. 6d). To further validate the PKN recruitment assay as a faithful readout of G_12/13_ activity, we assessed the effect of co-expressing either WT or constitutively active forms (CAM) of Gα_12_ or Gα_13_. As seen in Fig. 6e, expression of WT Gα_12_ and to a greater extent of CAM Gα_12_ and CAM Gα_13_ significantly increased recruitment of PKN to the plasma membrane. Finally, we designed a plasma membrane-targeted inhibitor of G_12/13_ activity by fusing the rgRGS domain of P115-RhoGEF (p115-RGS) to the membrane anchoring CAAX domain (p115-RGS-CAAX) (Fig. 6b). As shown in Fig. 6f, expression of p115-RGS-CAAX completely blocked the ability of U46619 to promote PKN recruitment to the plasma membrane as assessed by ebBRET^39^. The G_q_ inhibitor YM254890 did not affect the response, confirming that PKN recruitment resulted mainly from G_12/13_ activation. The selectivity of p115-RGS-CAAX inhibitory action on G_12/13_ was confirmed by its lack of effect on the activation of G_q_, G_i2_ or G_s_-mediated cAMP production stimulated by TPαR, D4R and β_1_AR, respectively (Supplementary Fig. S5b–d). Expression of the biosensor component did not influence the cell surface receptor expression (Supplementary Fig. S4c). As shown in Fig. 6g,h, stimulation of the β_1_AR promoted PKN recruitment to the plasma membrane that was blocked by the expression of the G_12/13_ activity dominant negative p115-RGS-CAAX, confirming engagement and activation of G_12_ by the β1AR.

### Calcium signalling

**Figure 7:**
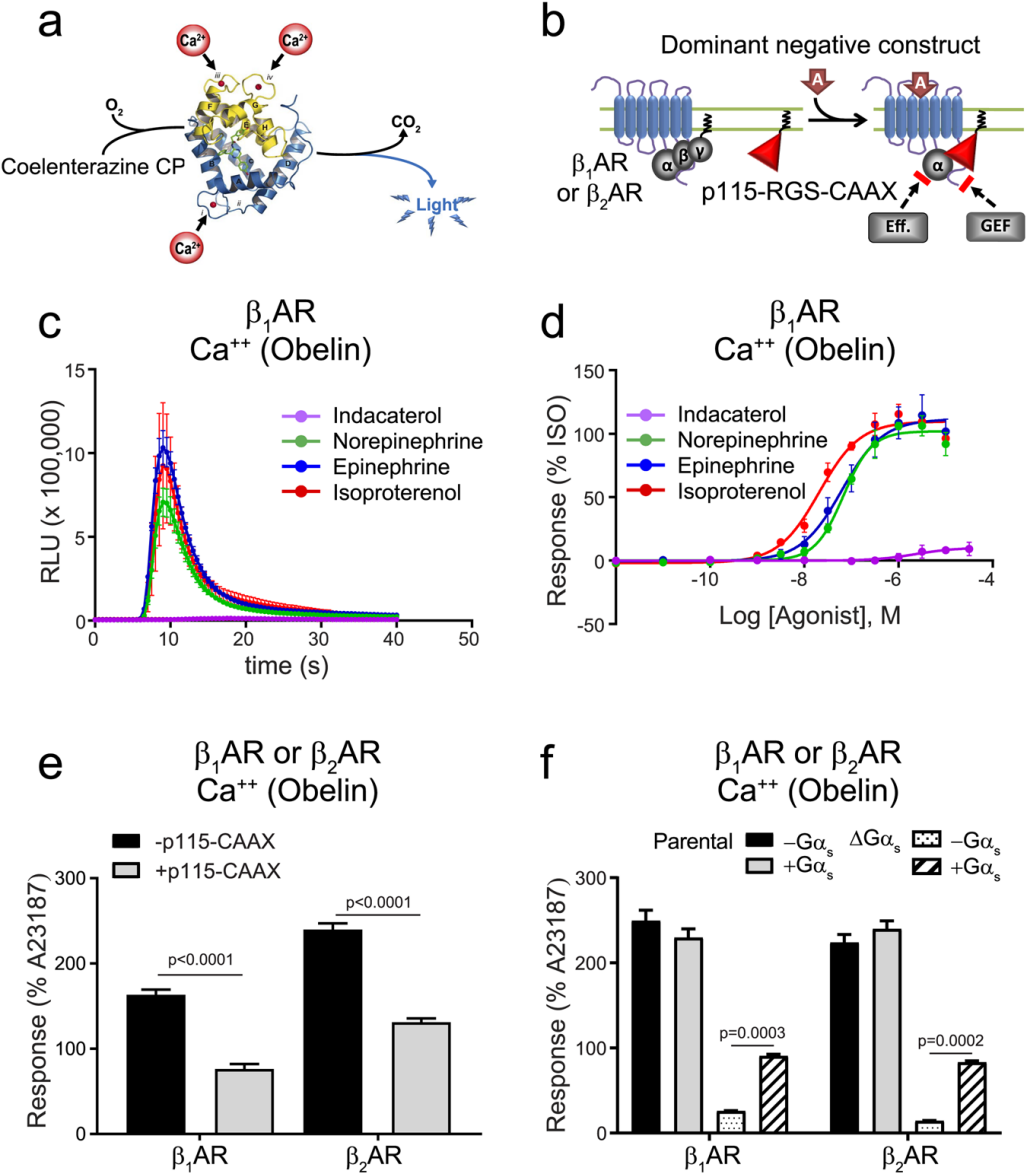
*Calcium response promoted by β*_1_*AR or β*_2_*AR activation*. (**a**) Schematic representation of the luminescence Obelin biosensor used to detect calcium. (**b**) Schematic representation of the mode of action of p115-RGS-CAAX (p115-CAAX) construct as a dominant negative construct for the inhibition of G_12/13_ signalling. HEK 293 cells stably expressing HA-β_1_AR were transfected with Obelin. (**c**) Kinetics of calcium response by indicated ligands (representative, isoproterenol, n = 10, other ligands n = 3) and expressed as relative luminescence units. (**d**) Concentration-responses curves for Ca^2+^ mobilization following β_1_AR activation by indicated ligands. Data were normalized to maximal isoproterenol response (100%), and expressed as mean ± SEM values. Detail of the number of experiments, maximal responses, pEC_50_ values and statistical comparisons of curve parameters for different ligands are provided in Supplementary Tables S1 and S2. (**e**) HEK 293 cells were transiently transfected with β_1_AR or β_2_AR along with the obelin biosensor, with or without the p115-CAAX inhibitor. Data were normalized to maximal A23187 response (100%), determined from the area under the curve (AUC), and are expressed as the mean ± SEM values (n = 3). Statistical comparisons were done using two-way ANOVA followed by post-hoc comparison with Tukey’s test. (**f**) Parental HEK 293 or ΔGαs cells were transfected with β_1_AR or β_2_AR, along with obelin, with or without Gα_s_. Data normalization and statistical analysis were done as described in (**e**) (n = 3).

Using a bioluminescent obelin calcium biosensor^40^, we observed that similar to β_2_AR activation^20^, β_1_AR stimulation leads to a calcium response^40^. As shown in Fig. 7, only three of the seven ligands tested (isoproterenol, epinephrine and norepinephrine) were able to stimulate a calcium response (Fig. 7c,d). Interestingly, these three compounds were also identified as full agonists using the G_12_ biosensor. Ligands such as isoetharine and indacaterol that were almost full agonists for the G_s_ pathway but only weakly activated G_12_ were unable to promote significant calcium mobilization, suggesting that G_12_ could play a role in the calcium response. To test this notion, we assessed βAR-mediated calcium mobilization in the presence or absence of the G_12/13_ dominant negative p115-RGS-CAAX construct. Isoproterenol-stimulated calcium mobilization through either βAR isoform (Fig. 7e) was blunted in the presence of p115-RGS-CAAX, confirming the functional relevance of G_12_ coupling. Again, both receptors were expressed at similar levels which was not altered by the presence of p115-CAAX (Supplementary Fig. S4d). Given that the receptor calcium responses were only partially affected by the inhibition of G_12/13_ signalling, we next examined potential contributions of other G proteins activated by these receptors. As shown in Fig. 7f, the isoproterenol-mediated response for either β_1_AR or β_2_AR was significantly reduced in CRISPR-Cas9-generated cells lacking Gα_s_^20^(ΔGs), again with no change in levels of receptor expression (Supplementary Fig. S4e). Restoration of G_s_ expression in the ΔGs cells rescued β_1_AR and β_2_AR-mediated calcium influx, confirming a role for G_s_ in this response. Taken together, the data indicate that both G_12/13_ and G_s_ contribute to βAR-mediated calcium mobilization.

### β-arrestin recruitment

**Figure 8:**
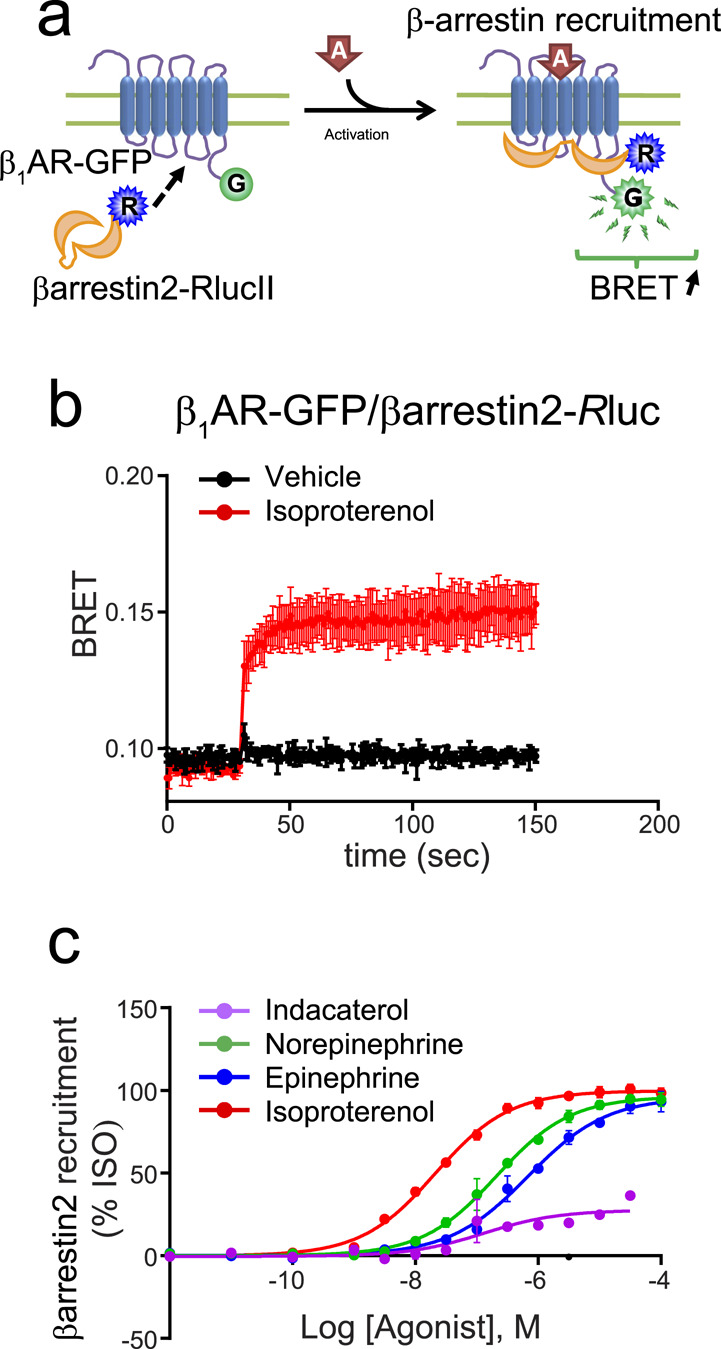
*β-arrestin2 recruitment by β*_1_*AR*. (**a**) Schematic representation of the βarr2 biosensor. HEK 293 cells were transfected with β_1_AR-GFP along with β-arrestin2-*R*luc. (**b**) Kinetic curves represent time course of β-arrestin2 recruitment (vehicle and isoproterenol n = 3) expressed as absolute BRET ratio. (**c**) Concentration-responses curves for β-arrestin2 recruitment following β_1_AR activation by indicated ligands. Data were normalized to maximal isoproterenol response, which was taken as 100%, and are expressed as mean ± SEM values. Detail of the number of experiments, maximal responses, pEC_50_ values and statistical comparisons of curve parameters for different ligands are provided in Supplementary Tables S1 and S2.

Finally, we examined recruitment of β-arrestin2 to the β_1_AR in response to various agonists using a previously described β-arrestin biosensor^41^ (Fig. 8). Interestingly, only isoproterenol, epinephrine and norepinephrine acted as full agonists, indacaterol being a partial agonist (Fig. 8c), while isoetharine, xamoterol and salmeterol showed no β-arrestin recruitment.

### Global signalling profiles

Given the granularity of the signalling profiles obtained, we analyzed the potential for functional selectivity amongst the pathways engaged by the β_1_AR. The operational model^42^ was used to capture signalling efficiency in the form of Log(τ/K_A_) (Supplementary Fig. S6, Supplementary Table S3). The propensity of each compound to activate the pathways considered was illustrated using a radial graph format in which each of the vertices represented activity towards one of the biosensors tested. Both maximal response and the efficiency expressed as Log(τ/K_A_) are shown. This analysis revealed that maximal response and efficiency profiles of isoproterenol, epinephrine or norepinephrine were practically identical for all the pathways analyzed. However, the patterns were quite different when considering the effects of isoetharine, salmeterol, indacaterol and xamoterol. These 4 compounds were partial agonists to different extents toward the G_s_/cAMP pathway and they displayed different abilities to promote detectable activation of G_i2_, G_z_, G_12_ and β-arrestin2. The most efficient of the four partial agonists toward G_s_/cAMP, indacaterol, was also able to recruit β-arrestin2, and activate Gα_12_, and Gα_z_. Isoetharine could activate G_12_ and G_s_, and salmeterol and xamoterol could only activate G_s_. These different signalling profiles are illustrated in Supplementary Fig. S7 in which the relative efficiency is color-coded for each of the signalling pathways studied. Whether this reflects true functional bias or pure partial agonism towards pathways coupled to the activated receptor is difficult to unambiguously determine. When comparing the efficiency of each ligand towards the different pathways using the operational model (log(τ/K_A_)), the rank order of coupling efficiency of each compound (except for xamoterol) was respected for all pathways measured (Supplementary Fig. S7). However, although indacaterol and xamoterol had similar efficiencies as epinephrine and norepinephrine to activate the G_s_/cAMP pathways, they were much weaker than these two compounds at activating G_i2_, G_z_, G_12_ or promoting the recruitment of β-arrestin2, in many cases not activating them at all. To a lesser extent, a similar comment can be made for isoetharine and salmeterol, although their efficiency to activate G_s_ and cAMP production is slightly less than norepinephrine and epinephrine. In any case, the data clearly reveals distinct signalling profiles for the different ligands that translate into distinct cellular outputs.

## Discussion

The β_1_AR was chosen here as prototypical GPCR where functional selectivity and biased signalling has been examined, albeit to a limited extent^7,8^. In previous studies, different ligands were reported to act in a biased fashion toward either ERK1/2 MAPK or adenylyl cyclase signalling and, in some cases antagonists or inverse agonists for the adenylyl cyclase pathway acted as agonists for the MAPK pathway. These studies and similar ones for other GPCRs^15,17,43^ open the possibility that fine-tuning receptor-specific signalling outputs with functionally selective ligands could generate superior therapeutic options for a number of diseases by disfavouring signalling cascades associated with adverse effects without compromising beneficial pathways. To do this would first require a broader approach to the signalosome downstream of the receptors to capture the ensemble of pathways that can be engaged by any given receptor. However, in most cases, only a limited number of pathways have been examined. In addition, comprehensive signal profiling of proximal and distal outputs can reveal novel signalling pathways downstream of a given receptor and how these pathways might interact. The approach proposed in the present study was to combine BRET-based signal profiling with genome editing and the use of pharmacological and genetically engineered inhibitors to allow a more detailed dissection of the relevant signalling architecture in a model cell type, establishing proof of principle.

G protein profiling was first used to identify novel signalling partners for the β_1_AR. Our data shows for the first time that both the β_1_AR and β_2_AR are coupled to the G_12_ signalling pathway. This was revealed not only by the activity of G_12_ itself, but also using novel biosensors detecting the engagement of Rho-GEF and the recruitment of PKN downstream of G_12_ activation, which was also found to contribute to βAR-stimulated calcium mobilization. Interestingly, our data suggest that neither receptor was coupled to G_13_, despite the fact that robust activation of G_13_ could be detected in response to TPαR activation using our biosensors. Such selectivity between the two members of the G_12/13_ family was also noted for GPR35^34^, which was found to be better coupled to G_13_ over G_12_. The implications for G_12_ signalling downstream of βAR activation may include cell shape changes as well as cell migration, mediated through activation of the Rho pathway.

Interestingly, we noted that both G_s_ and G_12_ seem to be involved in βAR-mediated calcium mobilization, suggesting a role in calcium signalling, beyond the well-characterized activation of voltage-gated calcium channels by βAR in excitable tissues^44–46^. β_2_AR-mediated calcium mobilization in non-excitable HEK 293 cells was previously reported to require transactivation of the purinergic P2Y receptor through a G_s_-dependent but cAMP-independent mechanism^20^. Whether G_12_ activation is also involved in this transactivation remains to be determined. For compounds such as isoproterenol that activated both G_s_ and G_12_, we found that the two pathways contributed to rise in cytoplasmic calcium, as cells deleted for either G_s_ or in which G_12/13_ was inhibited by the G_12/13_ dominant negative construct, p115-RGS-CAAX showed calcium responses significantly lower than those observed in the parental cells. Interestingly, compounds such as indacaterol, isoetharine and salmeterol that were strong partial agonists for G_s_ but weak partial agonist (indacaterol, isoetharine) or unable to activate G_12_ (salmeterol) could not generate significant calcium mobilization. This is consistent with an important role of G_12_ in calcium mobilization. Dominant-negative RhoA or the ROCK inhibitor Y-27632 were previously shown to block GPR55-mediated Ca^2+^ transients^47,48^, consistent with a role for the G_12/13_ pathway in calcium responses. In a recent paper, the β_1_AR was proposed to couple to G_14_ using DREADDs in combination with CRISPR gene deletions^39^. In theory, part of the calcium response could originate from this pathway as well. Although this cannot be excluded, this is unlikely since RNA-Seq analysis did not show detectable expression of G_14_ in the cells we used (Supplementary Table S4).

Another novel observation of our study is the subtype-selective coupling of β_1_AR to G_i_ family members. Although coupling to G_i_ had already been shown for the β_2_AR^30,31^, our study is the first report showing that the β_1_AR can also couple to G_i_ family members. When considering the pertussis toxin-sensitive G_i_ isoforms, β_1_AR-mediated activation could only be detected for G_i2_, indicating a possible selectivity among the G_i_ protein family members. This apparent selectivity did not result from a different sensitivity of the BRET biosensors themselves, since dopamine D2R-mediated activation of G_i1_ was more robust than that of G_i2_ while those of G_i3_, G_oA_ and G_oB_ were equivalent to G_i2_. Regardless of whether this also reflects functional selectivity for the D2R G_i/o_ subtypes engagement, our biosensors have the dynamic range to capture distinct levels of G protein activation by different GPCRs. Thus, as observed for the G_12/13_ family, β_1_AR can display selectivity among members of a same G protein subfamily. This observation may provide interesting avenues to further explore functional differences among different members of the same Gα subfamily and start to understand the evolutionary pressures that maintained these closely related subtypes.

Our data also reveal for the first-time robust coupling of both the β_1_- and β_2_AR to G_z_, a member of the G_i_ subfamily lacking an ADP ribosylation site, making them pertussis toxin-insensitive. This subtype has been shown to inhibit adenylyl cyclase types I, V and VI^49^ and has been found to have a very low intrinsic GTPase activity compared to other G protein subtypes, including other G_i_ isoforms. It is widely expressed in many tissues and is found in high levels in the central and peripheral nervous systems as well as in the platelets. In these circulating cells, G_z_ knockout mice were found to display abnormal platelet aggregation at physiological concentrations of epinephrine^50^. The physiological implications of this G_z_ coupling to the βARs remain to be investigated but the possibility of a counter regulatory action on the G_s_-stimulated production of cAMP in tissues expressing G_z_ and the adenylyl cyclase sensitive to this G_i_ subunit will be worth exploring. It should be noted that the potency of ligands to activate G_z_ is an order of magnitude lower than that for G_s_ activation, which would be consistent with a biphasic regulation of adenylyl cyclase, allowing for tight regulation and possibly oscillatory behaviours.

The present paper clearly illustrates that broader profiling of signalling pathways that can be engaged by a receptor can reveal signalling pathways that had not been anticipated before, even for receptors as well studied as the β_1_- and β_2_AR. We acknowledge that apparent differences in coupling efficacy between the β_1_- and β_2_AR could result from different expression levels, as they were not directly compared. The data also reveals that different ligands produce distinct signalling profiles leading to the activation to specific subsets of a given receptor’s repertoire. Whether these different profiles result from *bona-fide* biased signalling or are consequences of different level of partial agonism and different coupling strengths of the different effectors engaged is difficult to unambiguously determine at this point, especially when no bias factor can be calculated because of the absence of response for some of the pathways. Nevertheless, the selective activation of weakly coupled pathways seems to correlate with the overall relative efficacy of the compounds tested, indicating that partial agonism plays an important role in the apparent functional selectivity. However, whether the different signalling profiles result from biased signalling or partial agonism, the end result is that some ligands activate certain pathways but not others and that this is bound to have functional consequences. Many of our biosensors are based on overexpression of G protein subunits which may alter native receptor/G protein stoichiometries. An additional caveat must be considered when using gene deleted lines where rewiring of signalling pathways might occur. Resolving such issues will require a closer focus using distinct approaches.

What impact might the novel signalling pathways uncovered in the present study and the ligand-selective signalling profiles observed have on clinical indications where β-adrenergic agonists or antagonists are used will be an interesting area for further study. For this purpose, it will be critical to move biosensors into more physiologically relevant cell systems as the HEK 293 cell may not reflect important differences in signalling pathways in disease-relevant cells. Our results showed a limited functional bias for the various agonists tested among the different pathways tested downstream of the β_1_AR, but was able to robustly discriminate between partial and full agonists. Thus, as a homogenous platform, our approach allows a more global appreciation of the signalling profile of a given cell.

## Material and Methods

### Cell culture and transfection

HEK 293 and ΔGα cells were grown in Dulbecco’s modified Eagle’s medium (DMEM) supplemented with 10% fetal bovine serum, 100 U/ml penicillin/streptomycin at 37 °C in 5% CO_2_. The HA-β_1_AR HEK 293 stable cell line was maintained in complete medium supplemented with 1 μg/ml of puromycin. Except for G_s_ titration and concentration-response curves with β_1_AR, all transfections were carried out as follows: 48 h before the experiments, cells were transfected in suspension using polyethylenimine at 3:1 PEI/DNA ratio and seeded (~3×10^4^ cells/well) in 96-well/plates pre-treated with poly-D-lysine. Each of the expression vectors and biosensor constructs were diluted in PBS, and the total quantity of DNA was completed to 1 µg/row with salmon sperm DNA. For G_s_ titration and concentration-response curves, stable HA-β_1_AR cells were plated at 9000 cells/well in a poly-ornithine treated white 96-well plate the day before transfection and were transfected using PEI (2.5ug PEI:1ug DNA ratio) at 150 ng total DNA (i.e. biosensor constructs, HA-β_1_AR vector and empty pcDNA3.1 vector) per well. 24 h post-transfection, the medium was changed. The HA-β_1_AR HEK 293 stable cell line was used for all β_1_AR experiments, except for G_q_ and G_13_ saturation curves and when experiments were done in parental and ΔGα (ΔGα_s_) cell lines. β_1_AR-encoding plasmid was also added in the transfection mix for all experiments, except for EPAC and obelin biosensors, where native responses were measured.

### Signalling biosensors

*G protein activation biosensors*: G protein activation was measured by monitoring the separation of Gα from Gβγ subunits. Cells were transiently transfected with the receptor (HA-β_1_AR, HA-β_2_AR, HA-TPαR or D2R (250–300 ng) along with the BRET-based biosensors composed of the specific Gα-Rluc (i.e. Gα_s_-67RlucII (40 ng), Gα_z_-94RlucII (60–100 ng), Gα_12_-84RlucII (50 ng), Gα_i2_-loopRlucII (40 ng), GFP10-Gγ1 (200–250 ng) and stoichiometric amounts of the untagged Gβ_1_ subunit (100 ng)^51,52^. Gα_s_, Gα_q_, Gα_12_, Gα_13_, Gα_i1_, Gα_i2_, Gα_i3_, Gα_z_, Gα_oA_ and Gα_oB_ (20–60 ng) were used to generate BRET saturation curves. However, only the Gα proteins giving a sufficient signal above vehicle, when activated with isoproterenol, were further studied with additional ligands in kinetics and concentration-response experiments. For saturation and concentration-response experiments, BRET values were monitored 1 to 3 min after agonist addition, except for G_s_ and G_12_ where they were monitored 10 min after agonist addition.

*EPAC cAMP biosensor*: Another BRET-based biosensor was used to monitor changes in cytosolic cAMP was described previously^29^. This consisted of an N-terminal GFP10 with a 5 amino acid residue (GSAGT) linker to a mutated EPAC1 (ΔDEP; T781A; F782A) biosensor^53^ and a C-terminal-RlucII with a 5 residue linker (KLPAT)^29^. Stable HA-β_1_AR HEK 293 cells were transiently transfected with 50 ng of EPAC biosensor per row of a 96-well plate. For concentration-response experiments, BRET values were monitored 15 min after agonist addition.

*p115RhoGEF biosensor to monitor Gα*_12_ *activity*: A BRET-based biosensor composed of RGS-homology (RH) domain (amino acids 1–246) of p115RhoGEF fused to GFP10 and G_12_-84RlucII was used to measure Gα_12_ activity. Cells were transfected with 40 ng of G_12_-84RlucII, 500 ng of p115RhoGEF-GFP10 and 300 ng of receptor per row of a 96-well plate. For concentration response experiments, BRET was monitored 2 minutes after agonist addition.

*PKN biosensors to monitor Rho activation*: Upon receptor stimulation, activated G proteins promote Rho activation through RhoGEF recruitment to the plasma membrane. The activated Rho then recruits the *R*lucII-tagged effector (PKN) to the PM. Rho activity is monitored using the BRET between *R*lucII and a membrane-bound rGFP. The Rho binding domain of PKN1 was tagged with RlucII (BRET donor) to monitor its recruitment to the plasma membrane using enhanced bystander BRET with rGFP-CAAX (kRAS)^23^. To validate PKN recruitment by TPαR, HEK 293 cells were transfected in suspension with 10–20 ng of PKN-RlucII, 300 ng of CAAX-rGFP and increased concentration of TPαR (0–200 ng) per row of a 96-well plate. Cells were pre-treated with TPαR antagonist SQ29, 548 (1 µM) for 24 min and stimulated for 6 min with U46619 (100 nm) and 5 min of coelenterazine 400a before monitoring the BRET signal. Kinetic experiments were performed in HEK 293 cells using 10 ng of HA-β_1_AR, 1 ng of PKN-*R*lucII and 300 ng of CAAX-rGFP. Prolume purple (1 µM) was added for 6 min and BRET was measured following injection of isoproterenol (1 µM) for 30 sec at 37 °C. To confirm specificity of Rho activation, HEK 293 cells were co-transfected with or without constitutively active RhoA mutant Q63A (50 ng per row of a 96-well plate). To validate the effect of Gα_12_/Gα_1__3_ pathway on PKN recruitment to the plasma membrane, EE-tagged versions of Gα_12_, Gα_13_ and Gα_12_/Gα_13_ CAM mutants (Q231L and Q226L, respectively) (10 ng) were transfected along with 100 ng of TPαR, 0.5 ng of PKN-RLucII and 300 ng of CAAX-rGFP per row of a 96-well plate. Cells were stimulated for 6 min with either vehicle or U46619 (100 nM), and BRET values were collected using Prolume purple (1 µM final) as a substrate.

*β-arrestin recruitment to the receptor*: A BRET-based biosensor composed of hβarrestin2-RlucII and β_1_AR-GP10 was used to monitor β-arrestin recruitment to the receptor. HEK 293 cells were transfected with 60 ng of hβarrestin2-RlucII and 1 µg of β_1_AR-GFP10. For concentration-response experiments, BRET values were monitored 10 min after agonist addition.

*p*11*5-CAAX-based inhibitor of G protein-mediated Rho activation*. p115-CAAX is composed of the G protein binding domains (rgRGS) of p115RhoGEF, targeted to the plasma membrane using the prenylated polybasic sequence from kRAS. Upon receptor stimulation, p115-CAAX is recruited to activated G_12_ or G_13_, preventing recruitment of WT RhoGEF and thus activation of Rho. The rgRGS domain is known to promote GTPase activity, further inhibiting G protein-mediated signalling. To validate p115-CAAX as an inhibitor of G_12_/G_13_ pathway, HEK 293 cells were transfected with HA-TPαR (10 ng), PKN-RlucII (1 ng) and rGFP-CAAX (300 ng) along with p115-CAAX or ssDNA (mock). Cells were pre-treated with YM254890 or vehicle for 30 min at 37 °C and stimulated with TPαR agonist U46619 (100 nM) or vehicle for 6 min. For calcium mobilization experiments, HEK 293 cells were transfected with 500 ng of Obelin biosensor, 10 ng of HA-β_1_AR or HA-β_2_AR and 20 ng of p115-CAAX. Kinetic data for isoproterenol (1 µM) and A23187 (5 µM) were collected for 30 sec at 37 °C, with an integration time of 0.5 sec. To assess the effect of p115-CAAX on G_q_/G_i_ signalling, cells were transfected with 10 ng of receptor, 5 ng of Gα_q_/Gα_i2_-RlucII, 100 ng of Gβ_1_WT, 200 ng of GFP10-Gγ_1_ and 20 ng of p115-CAAX. BRET values were collected after 6 min of agonist stimulation (TPαR: U46619, D4R: dopamine) at 37 °C and addition of Prolume purple. The influence of p115-CAAX on cAMP response from endogenous β_2_AR or overexpressed β_1_AR (10 ng) was assessed using EPAC biosensor (25 ng). BRET values were collected after 10 min of Isoproterenol stimulation and addition of coelenterazine 400a, as described in calcium transfection (see below).

### BRET experiments

BRET was in general performed as previously decribed^29,51,52^. 48 h after transfection, cells were washed once with PBS and incubated for 1 h at 37 °C in Tyrode’s-HEPES buffer (137 mM NaCl, 0.9 mM KCl, 1 mM MgCl_2_, 11.9 mM NaHCO_3_, 5.5 mM glucose, 3.6 mM NaH_2_PO_4_, 25 mM HEPES, and 1 mM CaCl_2_, pH 7.4). The expression levels of the energy acceptor GFP10-tagged proteins were measured as total fluorescence using a FlexStation II microplate reader (Molecular Devices, Sunnyvale, CA, USA) with excitation at 400 nm and emission at 510 nm. Before β_1_AR activation, cells were exposed to ICI 118,551 (10 nM) for 30 min prior to the experiment in order to inhibit β_2_AR activity. For concentration-response experiments, cells were treated, with or without ligands, for the indicated times, and BRET was measured using a TriStar2 LB 942 Multidetection Microplate Reader (Berthold Technologies), equipped with a BRET400-GFP2/10 filter set (acceptor, 515 ± 20 nm; and donor, 400 ± 70 nm filters), 5 min after the addition of 2.5 µM of coelenterazine 400a. Absolute BRET signals (BRET) were derived from emissions detected with the energy acceptor filter divided by emission detected using the energy donor filter, while netBRET signals were obtained by subtracting BRET signals obtained in cells expressing the Rluc-fused donor constructs alone. ΔBRET was calculated by subtracting ligand-induced BRET from vehicle BRET. For kinetic experiments, cells were preincubated with coelenterazine 400a for 5 min, followed by readings for the indicated times, following addition of drugs. For G_s_ and G_12_ titration and concentration-response curves, 48 h post-transfection, wells were washed once with Kreb’s/HEPES buffer (146 mM NaCl, 4.2 mM KCl, 0.5 mM MgCl_2_, 1 mM CaCl_2_, 5.9 mM Glucose and 10 mM HEPES pH 7.4) and 80 μl of Kreb’s/HEPES buffer was added and plates left for 2–3 h at 37 °C. Then, 10 nM ICI 118,551 was added and plates left another 30 min at 37 °C to block endogenous β_2_AR. Finally, agonists were added and BRET assessed similarly as described above, using a FLUOstar Optima (BMG) equipped with BRET2 filter set (410 nm/515 nm). Saturation assays were performed initially to determine optimal donor to acceptor ratios for kinetic experiments and concentration-response curves and read 1 to 3 minutes after agonist addition (concentration 1 uM isoproterenol). Kinetic measurements were performed 48 h post-transfection, 5 minutes after addition of coelenterazine 400a (2.5 µM) or Prolume purple (1 µM, 6 min).

### Calcium mobilization

An obelin biosensor was used as a calcium reporter as described previously^6,54,55^. Stable HA-β_1_AR HEK 293 cells were transfected in suspension with 500 ng of WT obelin-pLVXi2H/per row. For experiments performed in parental and cells lacking Gα_s_ proteins (ΔG_s_), cells were transiently transfected with 100 ng of receptor and WT obelin. 48 h after transfection, cells were washed once with Tyrode’s-HEPES buffer and incubated with the obelin substrate, coelenterazine cp (1 µM; Biotium) for $\sim$2 h in the dark. For concentration-response experiments, increasing concentrations of agonists, diluted in Tyrode’s buffer, were injected into the wells and luminescence was measured using a SpectraMax L (Molecular Devices). Kinetics of activation were determined for each ligand concentration for 60 s and concentration-response curves were determined from the area under the curve (AUC). For Gα_S_ complementation experiments, HEK 293 T parental or cells lacking Gα_S_ were transfected with 10 ng of β_1_ or β_2_AR, 500 ng of obelin, in the presence or absence of 10 ng of Gα_S_. Luminescence was measured using a Mithras LB940 microplate reader.

### Cell surface ELISA

Parental HEK 293 cells or cells lacking Gα_s_ were transfected with HA-β_1_AR, HA-β_2_AR or HA-TPαR. 48 h after transfection cells were washed with PBS and fixed with 3% paraformaldehyde for 10 min at RT. Cells were labelled with mouse anti–HA-HRP antibody (3F10; 1:2000) for 1 h at RT in Wash B buffer (PBS supplemented with 0.5% BSA), followed by extensive washing (3×10 min). Vybrant DyeCycle Orange stain was added for 30 min at a final concentration of 10 μM at RT. Vybrant fluorescence was measured with excitation at 519 nm and emission at 563 nm using a FlexStationII microplate reader (Molecular Devices, Sunnyvale, CA, USA) to control for the number of cells/well. Total luminescence was measured 2 min after the addition of the HRP substrate Western lightning plus ECL using a Mithras LB940 microplate reader. Receptor cell surface expression was calculated as ratio of total luminescence to vibrant fluorescence.

### Data analysis

Concentration response curves describing ligand responses by different ligands were analyzed with Graphpad Prismv6 (GraphPad Software, La Jolla, CA), using built-in 3 or 4 parameter logistic equations to obtain independent pEC50 and maximal response values for different receptor-biosensor pairs *y* = a + (b-a)/(1 + 10^(logEC50-*x*)^*c) where: *y* is the measured response; *a* is the minimal asymptote, *b* is the maximal asymptote; *b-a* is maximal response and c is the slope.

Data were also fit with the operational model of Black and Leff^42,56^ using a set of equations kindly provided by Dr. Arthur Christopoulos. These equations were introduced into Graphpad Prism6:

$${\rm{A}}={10}^{{\rm{x}}}$$

$${\rm{operate}}1={((1+{\rm{A}}/(1))/(({10}^{{\rm{LogR}}})\ast {\rm{A}}))}^{{\rm{n}}}({\rm{used}}\,{\rm{to}}\,{\rm{fit}}\,{\rm{full}}\,{\rm{agonists}})$$

$${\rm{operate}}2={((1+{\rm{A}}/({10}^{{\rm{LogKA}}}))/(({10}^{{\rm{LogR}}})\ast {\rm{A}}))}^{{\rm{n}}}({\rm{used}}\,{\rm{to}}\,{\rm{fit}}\,{\rm{partial}}\,{\rm{agonists}})$$

$${\rm{Full}}\,{\rm{agonist}}={\rm{basal}}+({\rm{Emax}}-{\rm{basal}})/(1+{\rm{operate}}1)$$

$${\rm{Partial}}\,{\rm{agonist}}={\rm{basal}}+({\rm{Emax}}-{\rm{basal}})/(1+{\rm{operate}}2)$$

$${\rm{basal}}\to {\rm{response}}\,{\rm{observed}}\,{\rm{in}}\,{\rm{the}}\,{\rm{absence}}\,{\rm{of}}\,{\rm{agonist}}$$

$${\rm{Emax}}\to {\rm{maximal}}\,{\rm{response}}\,{\rm{of}}\,{\rm{the}}\,{\rm{system}}$$

$${\rm{n}}\to {\rm{slope}}\,{\rm{of}}\,{\rm{the}}\,{\rm{function}}\,{\rm{which}}\,{\rm{links}}\,{\rm{occupancy}}\,{\rm{to}}\,{\rm{response}}$$

$$KA\to {\rm{functional}}\,{\rm{affinity}}({\rm{partial}}\,{\rm{agonists}}).$$

A one-phase exponential decay was used to calculate the half-life in kinetic experiments. Statistical significance of ligand-induced changes at the different biosensors was established using one-way ANOVA to reveal concentration effects. pEC_50_ and maximal responses of different ligands were compared to corresponding isoproterenol values by means of one-way ANOVA followed by post-hoc Dunnett test. Specific details on statistical comparisons are provided in legends of Figures and Tables.

## Supplementary information

Supplemental information.

## Data Availability

The authors declare that all data supporting the findings in this study are presented within the article and its associated Supplementary Information Files. These are available from the corresponding authors upon request. Our biosensors are licensed to Domain Therapeutics for commercial purposes but are freely available to the academic community.
