# Supplementary material for: Signal profiling of the β1AR reveals coupling to novel signalling pathways and distinct phenotypic responses mediated by β1AR and β2AR
Source: Sci Rep. 2020 May 29;10:8779. doi: 10.1038/s41598-020-65636-3 (PMC7260363; doi:10.1038/s41598-020-65636-3)
Supplement: Supplementary file 1 — Supplemental information. [file 41598_2020_65636_MOESM1_ESM.docx]

**Signal profiling of the β_1_AR reveals coupling to novel signalling pathways and distinct phenotypic responses mediated by β_1_AR and β_2_AR**

Viktoriya Lukasheva^1§^, Dominic Devost^2§^, Christian Le Gouill^1§^, Yoon Namkung^3^, Ryan D. Martin^2^, Jean-Michel Longpré^4^, Mohammad Amraei^5^, Yuji Shinjo^6^, Mireille Hogue^1^, Monique Lagacé^1^, Billy Breton^1^, Junken Aoki^6^, Jason C. Tanny^2^, Stéphane A. Laporte^2,3^, Graciela Pineyro^5^, Asuka Inoue^6^, Michel Bouvier^1*^ and Terence E. Hébert^2*^

^1^Department of Biochemistry and Molecular Medicine, Institute for Research in Immunology and Cancer (IRIC), Université de Montréal, Montréal, Québec, Canada

^2^Department of Pharmacology and Therapeutics, McGill University, Montréal, Québec, Canada;

^3^ Department of Medicine, Research Institute of the McGill University Health Centre, McGill University, Montréal, Québec, Canada

^4^ Institut de Pharmacologie and Department of Pharmacology-Physiology, Faculty of Medicine and Health Sciences, Université de Sherbrooke, Sherbrooke, Canada

^5^ Department of Pharmacology and Physiology, Université de Montréal, Centre de Recherche de l’Hôpital Ste-Justine, Montréal, Canada

^6^Graduate School of Pharmaceutical Sciences, Tohoku University, Sendai, Japan

^7^Domain Therapeutics NA

^§^These authors contributed equally to this work

* Co-corresponding authors.

**Supplementary Materials and Methods**

**Materials**

(-)-Isoproterenol hydrochloride, (L)-norepinephrine (+)-bitartrate, (-)-epinephrine (+)-bitartrate, isoetharine mesylate were from Sigma-Aldrich. ICI 118,551 hydrochloride was from ApexBio or Tocris, while xamoterol hemifumarate, A23187, rotigotine hydrochloride and salmeterol xinafoate were from Tocris. Indacaterol maleic acid salt was from Toronto Research Chemicals Inc. U46619 and SQ-29,548 were from Cayman Chemical. YM254.890 was from WAKO while salmon sperm DNA and Vybrant® DyeCycle™ Orange stain were from Invitrogen. Prolume purple was from Nanolight Technology, coelenterazine 400a and CP were from Biotium. Polyethyleneimine, linear, MW.25.000 was from Polysciences or AlfaAesar. Monoclonal rat anti-HA-HRP antibody (clone 3F10) was from Roche or Sigma, while monoclonal mouse anti-HA.11 antibody was from Sigma-Aldrich and goat anti-mouse Alexa-Fluor 647 secondary antibody was from Molecular Probes.

**Receptor constructs**

A construct bearing the D2 dopamine receptor was obtained from cdna.org (Bloomsburg, PA) The human dopamine D4 receptor (D4R) coding sequence was synthesized at GeneART (Thermofisher) and subcloned in pCDNA3.1 Zeo(+) by Gibson assembly. HA-TPαR and β_1_AR-GFP10 constructs were described previously^1,2^. To generate the HA-tagged human β_1_AR construct, the β_1_AR coding sequence was PCR-amplified from pBC12BI hβ_1_AR using the following primers: 5’-GTGTTGGATCCGGCGCGGGGGTGCTCGTCCTG-3’ and 5’- CGCGGATCGTTTAAACGGGCCCTACACCTTGGATTCCGACGG-3’, then subcloned using BamHI + PmeI into pIRESpuro3 vector (Clontech Laboratories, CA) containing the coding sequence for the HA epitope tag. The HA-β_2_AR construct was generated by PCR amplification using the following primers: 5’-GTGTTGGATCCGGGCAACCCGGGAACGGCAGCG-3’ and 5’-AAAAGTAGAAAAACTGCTTTACAGCAGTGAGTC-3’. The PCR fragment was digested with BamHI and DraI restriction enzymes and subcloned into BamHI-PmeI restriction sites of the pIRESpuro3 HA vector.

**Biosensor constructs**

GFP10-Gγ1, Gβ1, hβArrestin2-RlucII, Gαs-67GFP10, Gα_i1_-loopRlucII, Gα_i2_-loopRlucII, Gα_i3_-loopRlucII, G_oA_-99RLucII, Go_B_-99RLucII, Gα_z_-94RlucII, EPAC db sensor, Gα_13_-130RlucII, Gα_q_-118RlucII, rGFP-CAAX, PKN-RlucII were described previously^2-10^. The constitutively active mutant Q63L RhoA was created by site-directed mutagenesis using PCR overlap method. The PCR fragment was cloned by Gibson assembly in pCDNA3.1 Zeo(+) digested with KpnI-HF+ EcoRI-HF. GαSL, Gα12EE, Gα13EE, G12αCAM (Q231L), G13αCAM (Q226L) were from cdna.org

*Gα_12_-84RlucII*: Gα12 coding sequence was PCR-amplified in 2 fragments: CMV-promoter-Gα12-EE-Nterm Primers: forward 5’- GATATACGCGTTGACATTGATTATTGAC-3’ & reverse 5’- TCGTACACCTTGCTGGTCATAGAACCACCACCACCGGATCCCTCGCGGCCGTGGATGATGCG-3’; Gα12-EE-Cterm Primers: forward 5’-AGAGTGCTGAAGAACGAGCAGTCTGGTGGTGGTGGATCTGGTACCTTCGACCAGAAGGCGCTGCTGGAG-3’ & reverse 5’- CGGGCCCTCTAGATCACTGCAGCATGATGTCCTTCAG-3’. The Rluc coding sequence was PCR-amplified from the EPAC db sensor construct using the following primers: 5’-CGCATCATCCACGGCCGCGAGGGATCCGGTGGTGGTGGTTCTATGACCAGCAAGGTGTACGA-3’ & 5’-CTCCAGCAGCGCCTTCTGGTCGAAGGTACCAGATCCACCACCACCAGACTGCTCGTTCTTCAGCACTCT-3’ The 3 fragments were assembled by PCR-overlap using the forward CMV-promoter-Gα12-EE-Nterm primer and the reverse-Gα12-EE-Cterm primer. PCR products were subcloned into pCDNA3.1 (+) using MluI+XbaI.

*pIRES hygro P115-GFP10-st2*: GFP10 was PCR-amplified from the EPAC db sensor construct using the following primers: 5’-GCTAGCGGATCCGCCGGTACCATGGTGAGCAAGGGCGAGGAG-3’ & 5’p-ATCGGATCCTTATTTTTCGAACTGCGGGTGGCTCCACTTGTACAGCTCGTCCATGCC-3’. PCR products were sub-cloned in pIRES Hygro3 (Clontech) using NheI + EcoRV. The RGS domain of P115 was PCR-amplified from an IMAGE clone (OpenBiosystems) using the following primers: 5’-GCGCGCTAGCATGGAAGACTTCGCCCGAGGGGCG-3’ & 5’-GGATCCGCTAGCCCGGAAGTTCCTCCCCGACTTCTTG-3’. PCR products were sub-cloned in pIRES Hygro3 GFP10st2 using NheI.

pLVXi-2H-*Obelin*: the obelin coding sequence was optimized, synthesized at GenScript and subcloned in pIRES Bleo (Clontech). Obelin was PCR-amplified using: 5’-CACACAGTCGACTAGTTCGAACCCGGGCGCGCCACCATGTCCAGTAAATATGCTGTCAA-3’ and 5’-CTAGCTTCTAGACCGGTTAACATGCATGTACACCAGCACACTGGTTTAAACAATTGACTCG-3’, digested with SalI + XbaI, then subcloned into pLVX IRES Hygro (Clontech) delta MfeI, digested with XhoI + XbaI. pLVX IRES Hygro delta MfeI was created by digesting with MfeI and Klenow.

*p115-CAAX G_12_/G_13_ inhibitor* To create pCDNA3.1 Zeo(+) p115RhoGEF (rgRGS)-CAAX, a construct encoding the RGS domain of P115 RhoGEF anchored to the plasma-membrane by the polybasic sequence and prenylation CAAX box of KRas, P115RhoGEF rgRGS cds, was PCR-amplified from pIRESH-p115-GFP10 using the following primers: P115-RGSL NheI Gibson FWD 5’-CTCACTATAGGGAGACCCAAGCTGGCTAGCCGCCACCATGGAAGACTTCGCCCGAGGG-3’ and P115-RGSL CAAX BamHI Gibson RVS 5’-GTGTTGTTGCTGGCCATGGTACCGGCGGATCCGCGGAAGTTCCTCCCCGACTTCTTGTCTCC-3’. The PCR fragment was subcloned by Gibson assembly in pCDNA3.1 Zeo(+) hrGFP-CAAX (ref: PMID: 27397672) digested with NheI+BamHI.

**Knock-out cell lines**

HEK293 parental (PL) and CRISPR-mediated Gα_s_ knockout (ΔGα_s_) cells were previously described^11^.

**Generation of HA-β_1_AR stable line.**

HEK 293 cells were transfected at 80% confluency with HA-β1AR in pIRESp3 vector. 48h after transfection, cells were detached using PBS/EDTA and labelled with monoclonal anti-HA.11 antibody followed by goat anti-mouse Alexa Fluor 647 secondary antibody in Tyrode’s buffer supplemented with 1% BSA. HA-positive cells were isolated using BDFACS ARIA II cell sorter, plated into 96-well plates and incubated in complete medium supplemented with puromycin for 2-3 weeks (puromycin 2 µg/ml, first week, 1 µg/ml for the next two, medium was changed twice a week). Several monoclonal and polyclonal cell lines were screened in functional assays using obelin and EPAC biosensors. One HA-β1AR stable cell line, insensitive to 10 nm ICI 118.551 in the obelin assay, was chosen for further analysis.

**RNA-Seq analysis**

HEK293 parental cells were maintained in Dulbecco’s Modified Eagle’s medium (DMEM) high glucose, 5% (v/v) fetal bovine serum and 1% (v/v) penicillin/streptomycin. Cells were plated at a density of 3x10^5^ cells per well in a 6 well plate (Thermo Scientific, 140675) and incubated for 24h prior to RNA isolation. RNA was isolated with the RNeasy® Mini Kit (Qiagen) according to manufacturer’s instructions. Libraries were prepared with the NEBNext rRNA-depleted (HMR) stranded library kit. Single-read 50bp sequencing was completed on the Illumina HiSeq4000 at the McGill University and Génome Québec Innovation Centre, Montréal, Canada. Reads were trimmed with the TrimGalore (0.6.0)^12,13^ wrapper with the following settings: --phred33 --length 36 -q 5 --stringency 1 -e 0.1. Following processing, reads were aligned to the human genome (GRCh38.95) with STAR (2.7.1a)^14^. Transcripts were assembled and RPKM values determined with StringTie (1.3.4d)^15^.

**References:**

1. Mercier, J.F., Salahpour, A., Angers, S., Breit, A. & Bouvier, M. Quantitative assessment of the beta 1 and beta 2-adrenergic receptor homo and hetero-dimerization by bioluminescence resonance energy transfer. *J Biol Chem* **277**, 44925-44931 (2002).

2. Parent, J.L., Labrecque, P., Orsini, M.J. & Benovic, J.L. Internalization of the TXA2 receptor alpha and beta isoforms. Role of the differentially spliced cooh terminus in agonist-promoted receptor internalization. *J Biol Chem* **274**, 8941-8 (1999).

3. Quoyer, J. et al. Pepducin targeting the C-X-C chemokine receptor type 4 acts as a biased agonist favoring activation of the inhibitory G protein. *Proc Natl Acad Sci U S A* **110**, E5088-97 (2013).

4. Gales, C. et al. Probing the activation-promoted structural rearrangements in preassembled receptor-G protein complexes. *Nat Struct Mol Biol* **13**, 778-86 (2006).

5. Richard-Lalonde M, N.K., Audet N, Sleno R, Amraei M, Hogue M, Balboni G, Schiller PW, Bouvie M, Hébert TE and Pineyro G. Conformational dynamics of Kir3.1/Kir3.2 channel activation via δ-opioid receptors (DORs). *Mol Pharmacol* **83**, 83:416–428 (2012).

6. Brule, C. et al. Biased signaling regulates the pleiotropic effects of the urotensin II receptor to modulate its cellular behaviors. *FASEB J* **28**, 5148-62 (2014).

7. Demeule, M. et al. Conjugation of a brain-penetrant peptide with neurotensin provides antinociceptive properties. *J Clin Invest* **124**, 1199-213 (2014).

8. Breton, B. et al. Multiplexing of multicolor bioluminescence resonance energy transfer. *Biophys J* **99**, 4037-46 (2010).

9. Namkung, Y. et al. Functional selectivity profiling of the angiotensin II type 1 receptor using pathway-wide BRET signaling sensors. *Sci Signal* **11**(2018).

10. Leduc, M. et al. Functional selectivity of natural and synthetic prostaglandin EP4 receptor ligands. *J Pharmacol Exp Ther* **331**, 297-307 (2009).

11. Stallaert, W. et al. Purinergic Receptor Transactivation by the beta2-Adrenergic Receptor Increases Intracellular Ca(^2+^) in Nonexcitable Cells. *Mol Pharmacol* **91**, 533-544 (2017).

12. Krueger, F. Trim Galore. *Available from:* [*http://www.bioinformatics.babraham.ac.uk/projects/trim_galore/*](http://www.bioinformatics.babraham.ac.uk/projects/trim_galore/)*.*

13. Martin, M. Cutadapt removes adapter sequences from high-throughput sequencing reads. *EMBnet* **17**, 10-12 (2011).

14. Dobin, A. et al. STAR: ultrafast universal RNA-seq aligner. *Bioinformatics* **29**, 15-21 (2013).

15. Pertea, M. et al. StringTie enables improved reconstruction of a transcriptome from RNA-seq reads. *Nat Biotechnol* **33**, 290-5 (2015).

16. Kenakin, T., Watson, C., Muniz-Medina, V., Christopoulos, A. & Novick, S. A simple method for quantifying functional selectivity and agonist bias. *ACS Chem Neurosci* **3**, 193-203 (2012).

17. Black, J.W. & Leff, P. Operational models of pharmacological agonism. *Proc R Soc Lond B Biol Sci* **220**, 141-62 (1983).


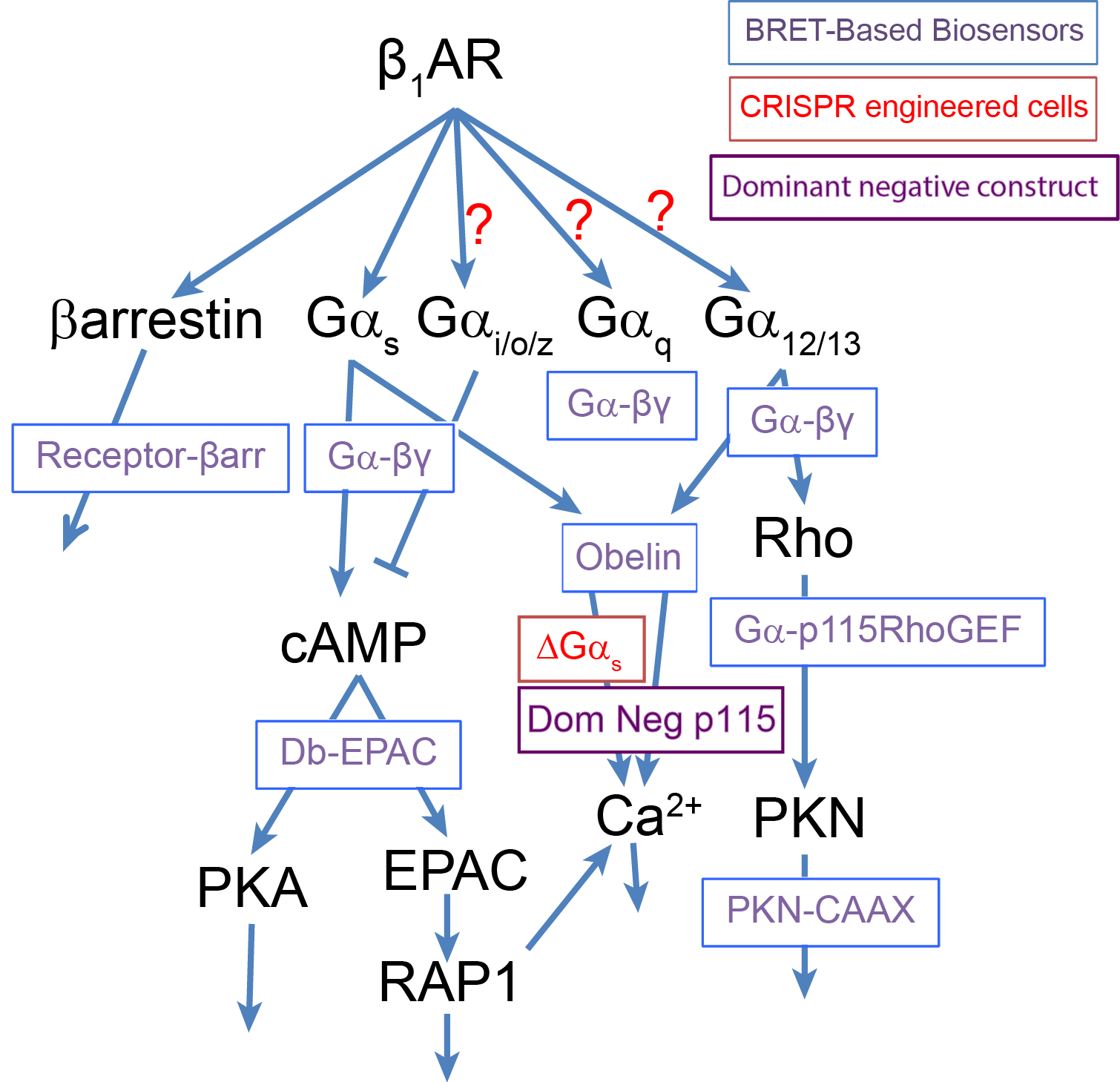


**Supplementary Figure S1**. *BRET-based biosensors and genome editing approaches used in this study*. Schematic representation of the different β_1_AR downstream pathways and effectors analyzed in this study. The BRET-based biosensors are presented in blue, dominant negative constructs in purple, while the CRISPR engineered cell lines are in red. The blue arrows represent an activation while blunted arrows show inhibition of the pathway. Question marks indicate potential β_1_AR signalling pathways that were investigated here for the first time.

**
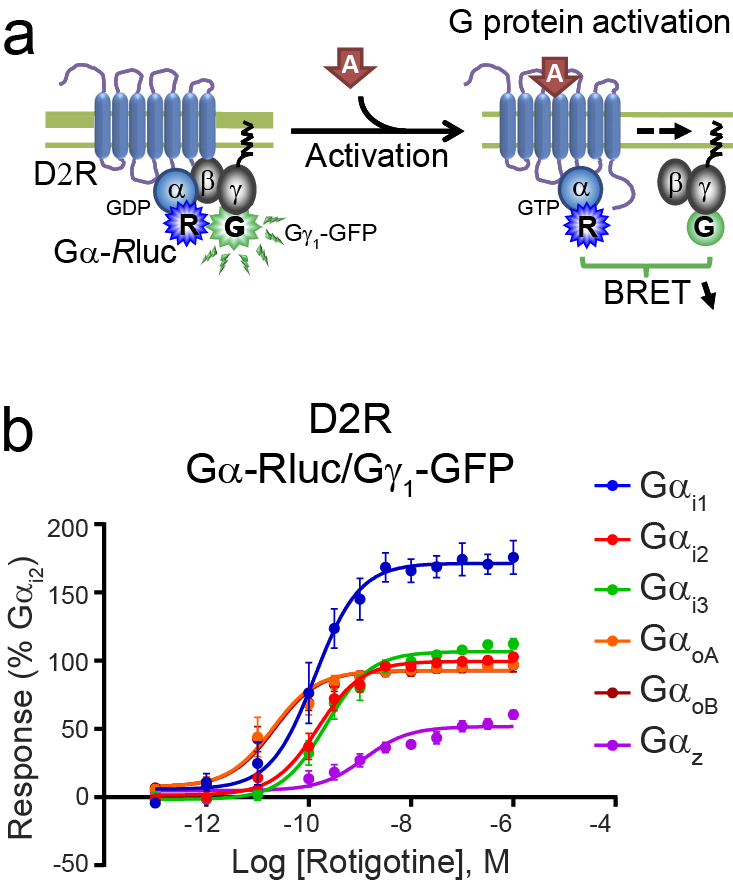
**

**Supplementary Figure S2**. *Identification of the Gα proteins involved in dopamine D2 receptor (D2R) signalling*. (**a**) Schematic representation of the Gα-Rluc/Gγ-GFP biosensor used to identify the Gα proteins involved in D2R signalling. (**b**) HEK 293 cells were transfected with the D2R, Gα-*R*luc (Gαi_1_, Gα_i2_, Gα_i3_, Gα_oA_ and Gα_oB_ and Gα_z_), Gγ_1_-GFP and untagged Gβ_1_. Concentration-response curves following D2R activation by rotigotine. Data were normalized to maximal Gα_i2_ response (100%) and are expressed as mean ± SEM of 3 independent experiments performed in duplicate.

**
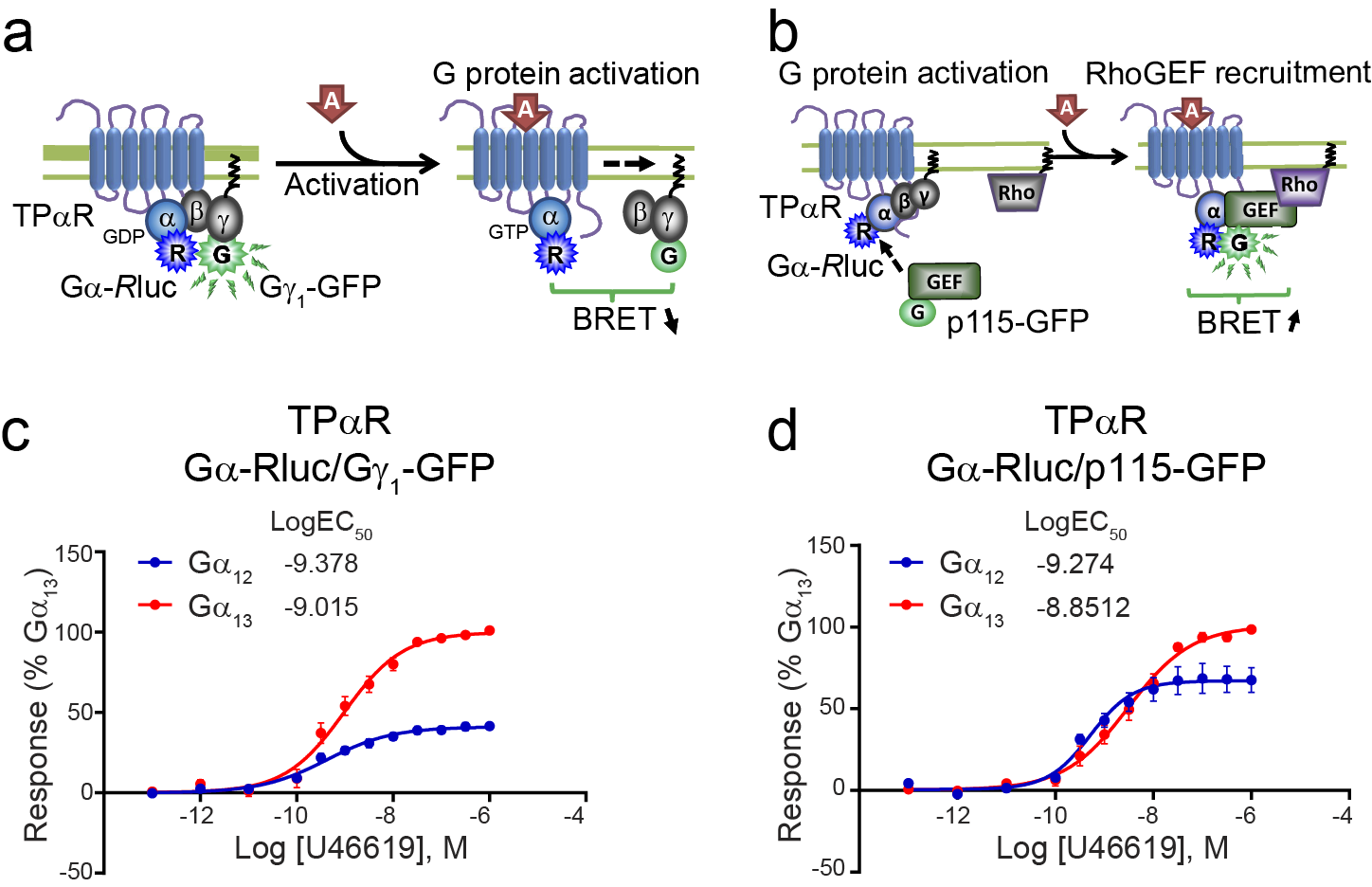
**

**Supplementary Figure S3**. *Gα_12/13_-induced activation by TPαR*. (**a**) Schematic representation of the Gα_12/13_ activation. (**b**) Schematic representation of the Gα_12/13_-p115 biosensors. HEK 293 cells were transfected with TPαR, along with (**c**) Gα-*R*luc (Gα_12_ or Gα_13_), Gγ_1_-GFP and untagged Gβ_1_(C), or (**d**) with Gα-*R*luc (Gα_12_ or Gα_13_) and p115-GFP. Concentration-response curve for (**c**) Gα_12/13_ activation or (**d**) Gα_12/13_-p115 biosensors activation upon Gα_12_ or Gα_13_ overexpression. Data were normalized to maximal Gα_13_ response (100%) and are expressed as mean ± SEM of (**c**) 3 or (**d**) 5 independent experiments performed in duplicate.

**
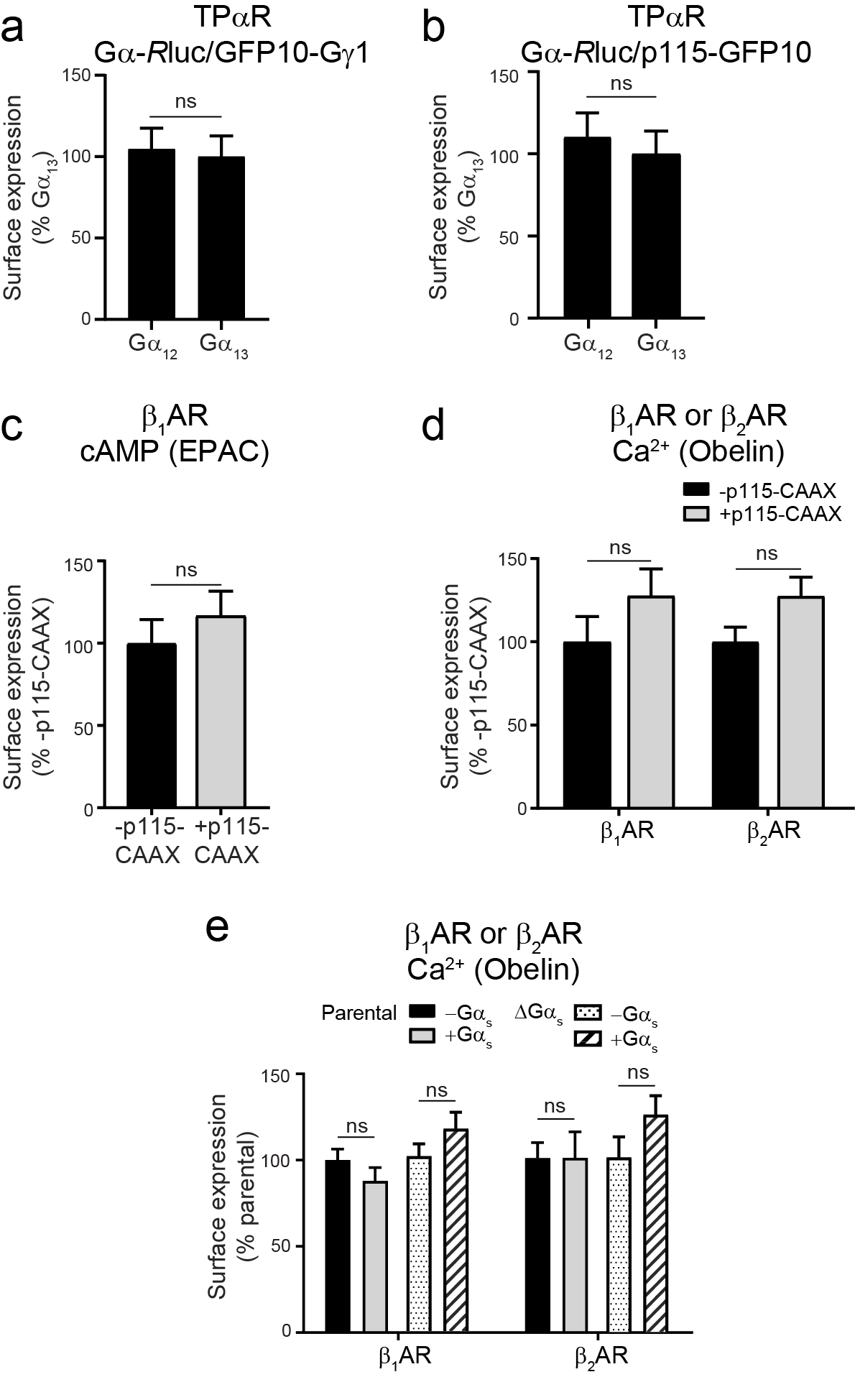
Supplementary Figure S4**. *Surface expression of receptors.* Receptor cell surface expression level in experiments where different partners were transfected together: (**a**) TPαR with Gα-Rluc and Gγ_1_-GFP10, with or without Gα_12_ or Gα_13_ (**Supplementary Fig. S3c**), (**b**) TPαR with Gα-Rluc and p115-Rho-Gef-GFP10 (p115-GFP10) (**Supplementary Fig. S3d**), (**c**) β_1_AR with cAMP (EPAC), with or without p115-RGS-CAAX (p115-CAAX) (**Supplementary Fig. S5d**), (**d-e**) β_1_AR or β_2_AR with Obelin, with or without p115-CAAX (HEK 293 cells, **Fig. 7e** and Parental vs ΔGαs cells, **Fig. 7f**). Receptor expression levels were measured using a mouse anti-HA-HRP antibody with an ELISA assay (see *Materials and Methods*). Data were normalized to maximal response (100%) of (**a-b**) Gα_13_, (**c-d**) -p115-CAAX or (**e**) non-transfected parental response, and are expressed as mean ± SEM ((**a,d,e**) n=3, (**c**) n=4 or (**b**) n=5). Statistical comparisons were done using (**a-d**) unpaired t test or (**e**) two-way ANOVA followed by post-hoc comparison with Tukey’s test. ns: non-significant.

**
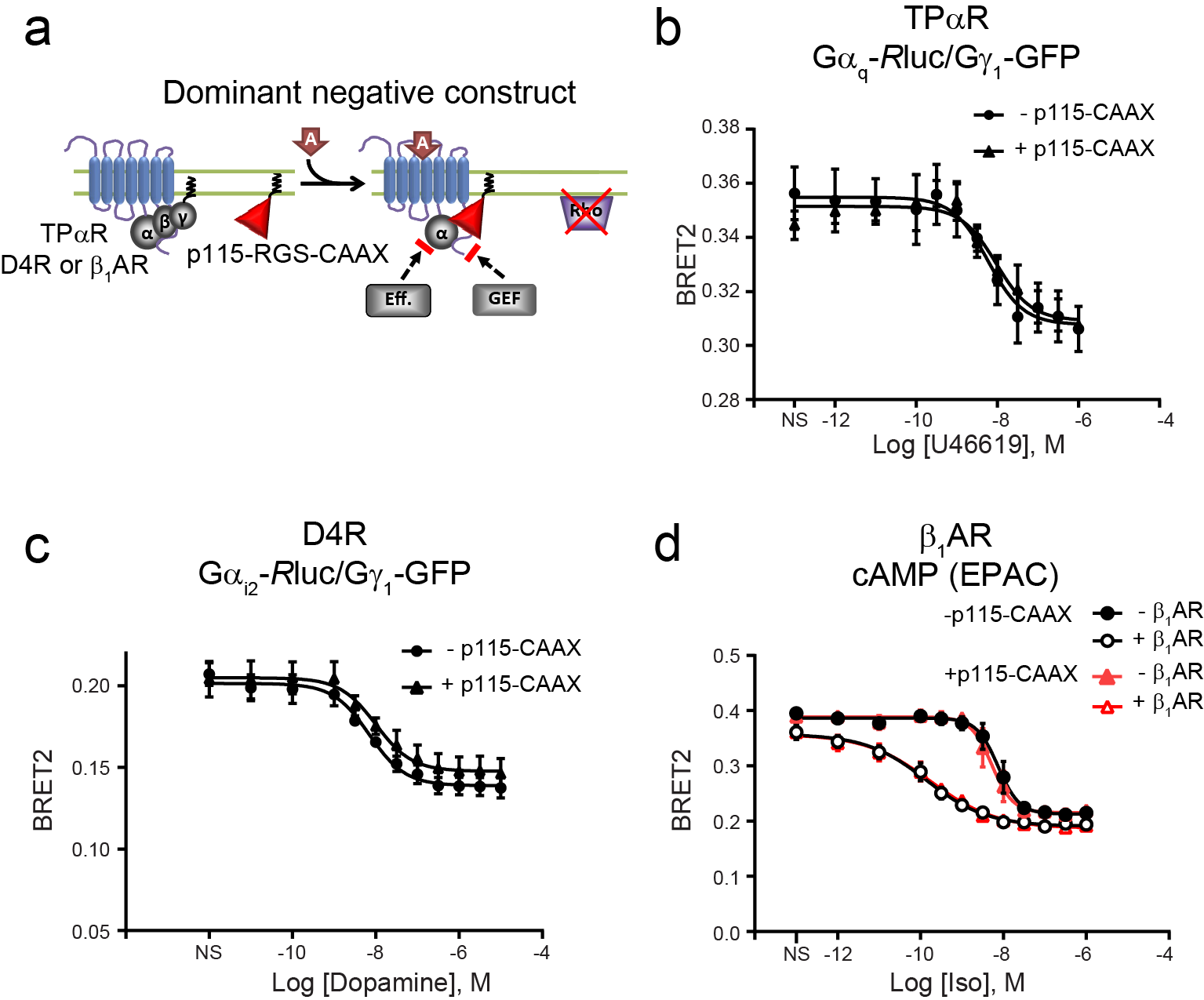
**

**Supplementary Figure S5.** *Characterization of* *p115-RGS-CAAX as a dominant negative construct for the G protein-mediated activation of the Rho signalling pathway.* (**a**) Schematic representation of the mode of action of p115-RGS-CAAX (p115-CAAX) construct. HEK 293 cells were transfected with (**b**) TPαR and Gα_q-_RlucII, GFP10-Gγ_1_ and Gβ_1_, (**c**) D4R and Gα_i2-_RlucII, GFP10-Gγ_1_ and Gβ_1_ or (**d**) β_1_AR and cAMP sensor (EPAC), with or without p115-CAAX. Dose response curve of (**b**) TPαR-induced Gα_q_ activation, (**c**) Dopamine D4R-induced Gα_i2_ activation or (**d**) β_1_AR-induced cAMP production are expressed as BRET signal and are the mean ± SEM ((**b-c**) n=3 or (**d**) n=4). A decrease in BRET reflects either (**b**) Gq or (**c**) Gα_i2_ activation, or (**d**) an increase in cAMP levels.


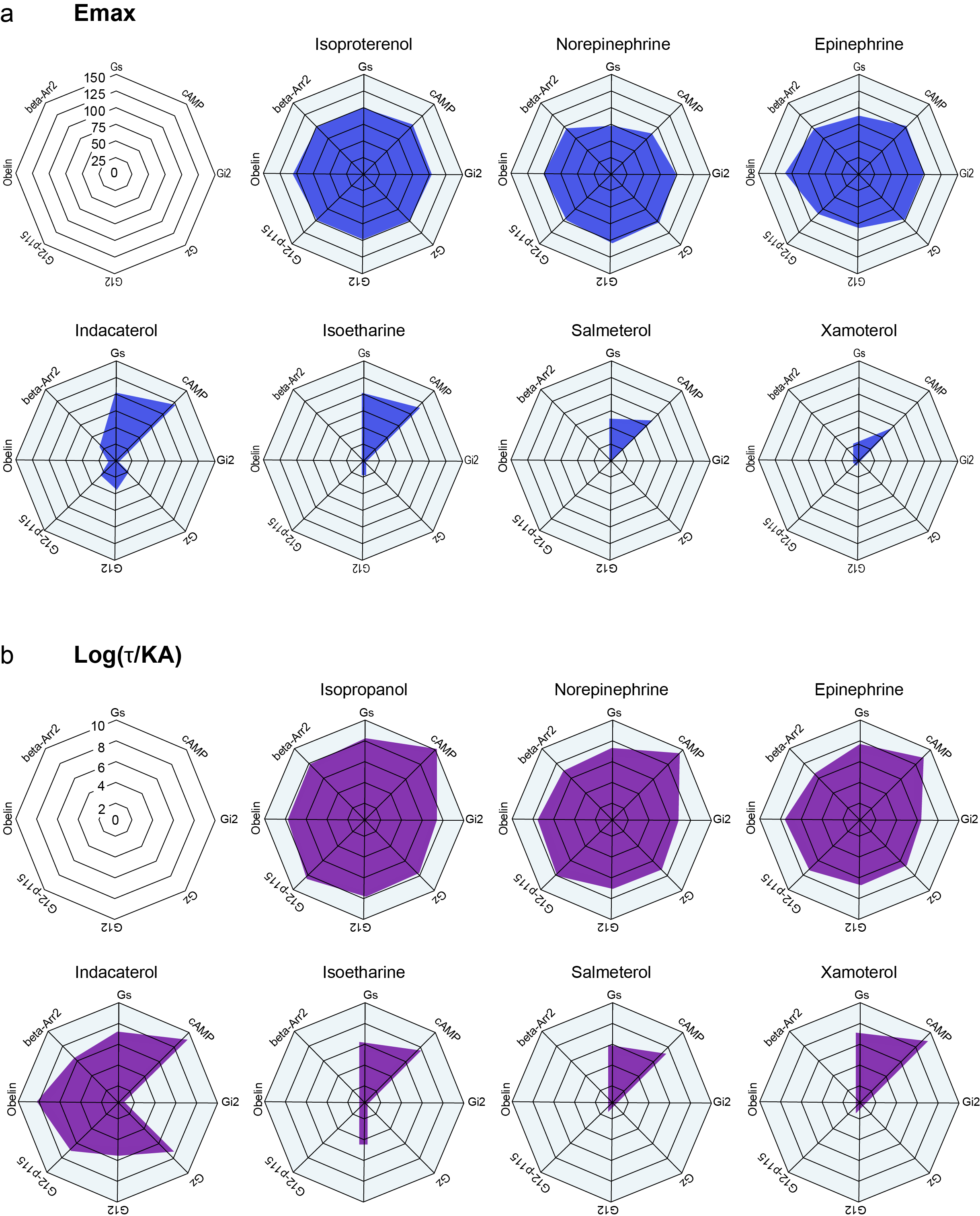


**Supplementary Figure S6**. *Graphic representation of ligand responses at β_1_AR*. **(A)** Logistic Emax values and (**B)** operational transduction coefficients (Log(τ/K_A_)) derived from concentration response curves generated by *β*_1_AR at nine different biosensors were represented as radial graphs. Each radius corresponds to the magnitude of Emax and Log(τ/K_A_) in the corresponding biosensor, as indicated. Transduction coefficients are shown using a logarithmic scale and Emax values, normalized to maximal isoproterenol responses, are presented on a linear scale. The key specified in the empty graph applies to all ligands and shows the order in which information for each biosensor is provided.

**
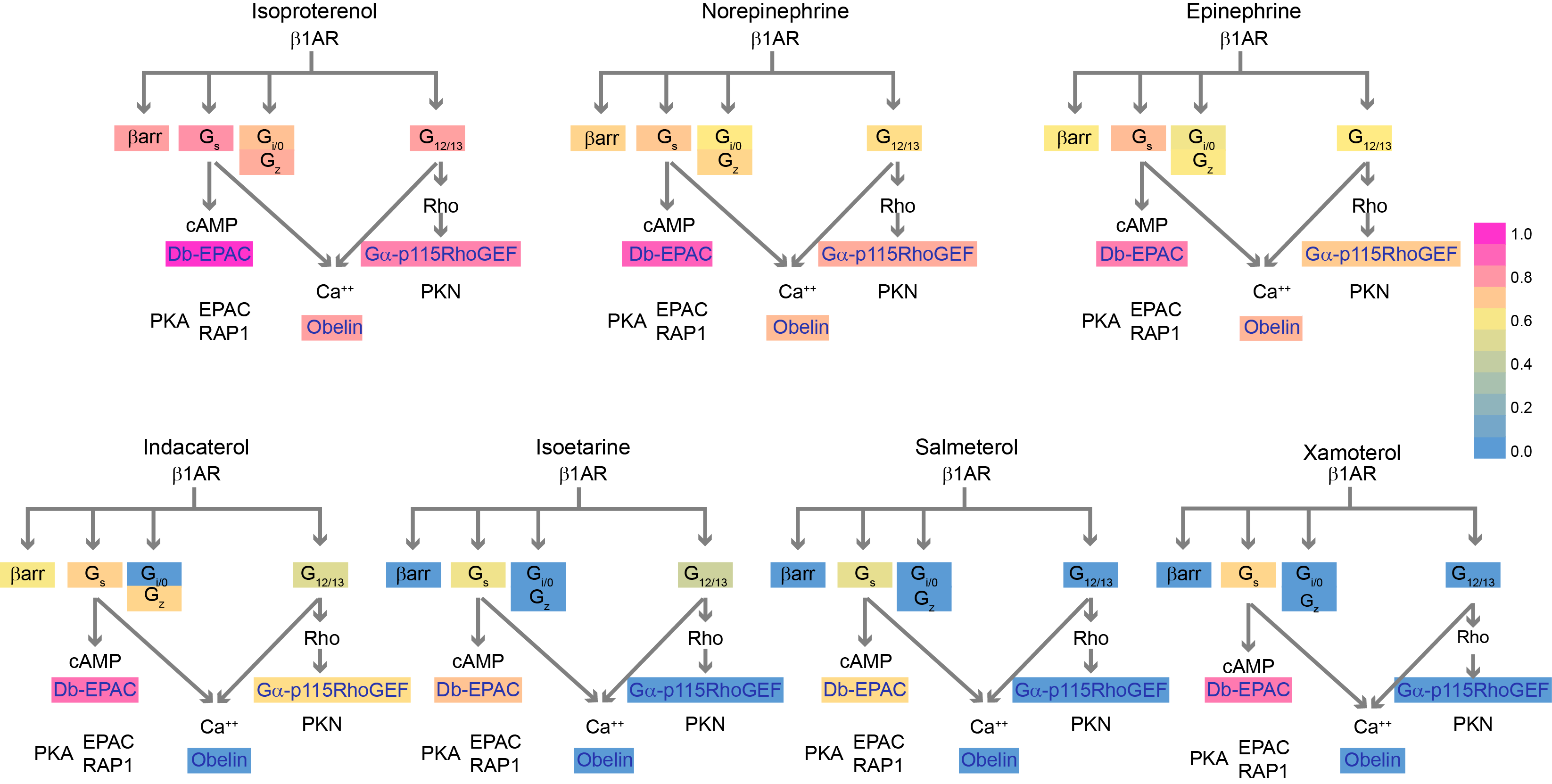
**

**Supplementary Figure S7**. *Ligand bias for β_1_AR signalling*. Signalling pathways for the indicated ligands illustrate color-coded weight of different signals in relation to Log(τ/K_A_) for the cAMP response elicited by isoproterenol.

**Supplementary Table S1:** *Maximal responses produced* *by clinically relevant adrenergic ligands at different signalling pathways engaged by β_1_AR.*

|  | Gs | | | | |  | cAMP (EPAC) | | | | |
| --- | --- | --- | --- | --- | --- | --- | --- | --- | --- | --- | --- |
|  | Maximal response | CI-95 | n | [Ligand] effect | Comparison ISO |  | Maximal response | CI-95 | n | [Ligand] effect | Comparison ISO |
| Isoproterenol | 100.60 ± 2.85 | 94.93 - 106.30 | 8 | <0.0001 |  |  | 106.50 ± 2.77 | 101.00 - 112.00 | 6 | <0.0001 |  |
| Epinephrine | 86.53 ± 5.64 | 75.00 - 98.06 | 4 | <0.0001 | 0.1747 |  | 100.80 ± 2.54 | 95.63 - 106.00 | 3 | <0.0001 | 0.8680 |
| Norepinephrine | 72.73 ± 3.06 | 66.42 - 79.05 | 3 | <0.0001 | 0.0038 |  | 85.20 ± 3.45 | 78.16 - 92.24 | 3 | <0.0001 | 0.0093 |
| Isoetharine | 95.67 ± 7.68 | 79.75 - 111.60 | 3 | <0.0001 | 0.9691 |  | 107.30 ± 6.54 | 94.13 - 120.50 | 4 | <0.0001 | 0.9998 |
| Indacaterol | 89.83 ± 9.93 | 69.19 - 110.50 | 3 | 0.0141 | 0.5203 |  | 105.60 ± 7.01 | 91.24 - 119.90 | 3 | <0.0001 | 0.9998 |
| Salmeterol | 55.78 ± 5.53 | 44.35 - 67.21 | 3 | <0.0001 | <0.0001 |  | 76.50 ± 3.27 | 69.90 - 83.11 | 4 | <0.0001 | 0.0001 |
| Xamoterol | 27.85 ± 3.75 | 20.10 - 35.60 | 3 | 0.0023 | <0.0001 |  | 65.26 ± 1.78 | 61.64 - 68.88 | 3 | <0.0001 | <0.0001 |
|  | Gi2 | | | | |  | Gz | | | | |
|  | Maximal response | CI-95 | n | [Ligand] effect | Comparison ISO |  | Maximal response | CI-95 | n | [Ligand] effect | Comparison ISO |
| Isoproterenol | 103.10 ± 2.96 | 97.14 - 109.00 | 5 | <0.0001 |  |  | 99.15 ± 1.44 | 96.25 - 102.00 | 4 | <0.0001 |  |
| Epinephrine | 97.85 ± 5.34 | 87.13 - 108.60 | 5 | <0.0001 | 0.6295 |  | 96.82 ± 2.74 | 91.30 - 102.30 | 4 | <0.0001 | 0.7772 |
| Norepinephrine | 96.62 ± 4.78 | 87.04 - 106.20 | 5 | <0.0001 | 0.5068 |  | 99.88 ± 2.25 | 95.35 - 104.40 | 4 | <0.0001 | 0.9891 |
| Isoetharine | No Response |  |  |  |  |  | No Response |  |  |  |  |
| Indacaterol | No Response |  |  |  |  |  | 20.54 ± 1.73 | 17.04 - 24.03 | 4 | <0.0001 | <0.0001 |
| Salmeterol | No Response |  |  |  |  |  | No Response |  |  |  |  |
| Xamoterol | No Response |  |  |  |  |  | No Response |  |  |  |  |
|  | G12 | | | | |  | G12-p115 | | | | |
|  | Maximal response | CI-95 | n | [Ligand] effect | Comparison ISO |  | Maximal response | CI-95 | n | [Ligand] effect | Comparison ISO |
| Isoproterenol | 98.88 ± 3.99 | 90.93 - 106.80 | 9 | <0.0001 |  |  | 99.69 ± 2.29 | 95.03 - 104.30 | 3 | <0.0001 |  |
| Epinephrine | 81.48 ± 6.54 | 68.10 - 94.86 | 4 | <0.0001 | 0.1581 |  | 86.46 ± 5.00 | 76.27 - 96.66 | 3 | <0.0001 | 0.0520 |
| Norepinephrine | 103.50 ± 9.10 | 84.67 - 122.30 | 3 | <0.0001 | 0.9712 |  | 97.58 ± 2.20 | 93.09 - 102.10 | 3 | <0.0001 | 0.9395 |
| Isoetharine | 21.56 ± 6.45 | 8.392 - 34.74 | 4 | 0.0389 | <0.0001 |  | No Response | No Response |  |  |  |
| Indacaterol | 34.05 ± 7.38 | 19.06 - 49.04 | 5 | 0.0494 | <0.0001 |  | 25.46 ± 2.81 | 19.74 - 31.18 | 3 | <0.0001 | <0.0001 |
| Salmeterol | No Response |  |  |  |  |  | No Response |  |  |  |  |
| Xamoterol | No Response |  |  |  |  |  | No Response |  |  |  |  |
|  | Ca^2+^ (Obelin) | | | | |  | βarrestin2 | | | | |
|  | Maximal response | CI-95 | n | [Ligand] effect | Comparison ISO |  | Maximal response | CI-95 | n | [Ligand] effect | Comparison ISO |
| Isoproterenol | 107.80 ± 2.79 | 102.10 - 113.50 | 3 | <0.0001 |  |  | 99.73 ± 1.24 | 97.23 - 102.20 | 4 | <0.0001 |  |
| Epinephrine | 110.40 ± 5.84 | 98.47 - 122.30 | 3 | <0.0001 | 0.9469 |  | 95.97 ± 3.71 | 88.53 - 103.40 | 4 | <0.0001 | 0.9911 |
| Norepinephrine | 101.90 ± 3.22 | 95.35 - 108.50 | 3 | <0.0001 | 0.5597 |  | 96.30 ± 2.45 | 91.38 - 101.20 | 4 | <0.0001 | 0.9938 |
| Isoetharine | No Response |  |  |  |  |  | No Response |  |  |  |  |
| Indacaterol | 8.66 ± 1.84 | 4.88 - 12.44 | 3 | 0.0761 | <0.0001 |  | 38.76 ±17.12 | 4.32 - 73.21 | 4 | <0.0001 | 0.0003 |
| Salmeterol | No Response |  |  |  |  |  | No Response |  |  |  |  |
| Xamoterol | No Response |  |  |  |  |  | No Response |  |  |  |  |

HA-β_1_AR-HEK 293 cells were stimulated with increasing concentrations of the indicated ligands. Concentration-dependent changes in BRET for each of the indicated ligands were expressed as maximal change induced by isoproterenol at each of the indicated signalling pathways and curves were fit with logistic 3 or 4 parameter equations. Maximal response corresponds to the asymptote expressed as mean ± SEM and corresponding confidence intervals (CI-95). Maximal response by each ligand was compared to that of isoproterenol using one-way ANOVA followed by Dunnett’s test. The p values for these comparisons appear in the “comparison ISO” column. BRET changes elicited by each ligand at different concentrations were analyzed by one-way ANOVA to establish concentration effects and the resulting p values appear under the column [Ligand] effect. “n” corresponds to the number of independent experiments for the indicated ligand and biosensor. Data were derived from experiments presented in **Fig. 2e** (Gα_s_), Fig. 2f (cAMP (EPAC)), **Fig. 3c** (Gα_i2_), **Fig. 4d** (Gα_z_), **Fig. 5e** (Gα_12_), **Fig. 5f** (Gα_12_-p115), **Fig. 7c** (Ca^2+^ (Obelin)) and **Fig. 8c** (β-arrestin2).

**Supplementary Table S2:** *Potencies (pEC_50_) of clinically relevant adrenergic ligands for distinct signalling pathways promoted by β_1_AR.*

|  | Gs | | | |  | cAMP (EPAC) | | | |
| --- | --- | --- | --- | --- | --- | --- | --- | --- | --- |
|  | pEC50 | CI-95 | n | Comparison ISO |  | pEC50 | CI-95 | n | Comparison ISO |
| Isoproterenol | 8.16 ± 0.10 | 7.96 - 8.35 | 8 |  |  | 9.97 ± 0.16 | 9.65 - 10.29 | 6 |  |
| Epinephrine | 7.51 ± 0.22 | 7.06 - 7.95 | 4 | 0.0999 |  | 8.42 ± 0.07 | 8.27 - 8.56 | 3 | <0.0001 |
| Norepinephrine | 7.45 ± 0.13 | 7.17 - 7.72 | 3 | 0.1063 |  | 9.27 ± 0.17 | 8.93 - 9.61 | 3 | 0.0253 |
| Isoetharine | 5.82 ± 0.19 | 5.43 - 6.21 | 3 | <0.0001 |  | 6.80 ± 0.16 | 6.47 - 7.12 | 4 | <0.0001 |
| Indacaterol | 6.86 ± 0.30 | 6.24 - 7.47 | 3 | 0.0011 |  | 8.65 ± 0.21 | 8.23 - 9.07 | 3 | <0.0001 |
| Salmeterol | 6.08 ± 0.25 | 5.57 - 6.59 | 3 | <0.0001 |  | 6.77 ± 0.09 | 6.58 - 6.95 | 4 | <0.0001 |
| Xamoterol | 8.10 ± 0.45 | 7.17 - 9.03 | 3 | 0.9997 |  | 8.95 ± 0.16 | 8.63 - 9.28 | 3 | 0.0010 |
|  | Gi2 | | | |  | Gz | | | |
|  | pEC50 | CI-95 | n | Comparison ISO |  | pEC50 | CI-95 | n | Comparison ISO |
| Isoproterenol | 7.00 ± 0.07 | 6.86 - 7.15 | 5 |  |  | 7.42 ± 0.04 | 7.34 - 7.51 | 4 |  |
| Epinephrine | 5.77 ± 0.08 | 5.61 - 5.93 | 5 | <0.0001 |  | 6.13 ± 0.06 | 6.02 - 6.25 | 4 | 0.0001 |
| Norepinephrine | 6.27 ± 0.09 | 6.08 - 6.46 | 5 | <0.0001 |  | 6.59 ± 0.06 | 6.48 - 6.71 | 4 | 0.0043 |
| Isoetharine | No Response |  |  |  |  | No Response |  |  |  |
| Indacaterol | No Response |  |  |  |  | 7.30 ± 0.28 | 6.74 - 7.85 | 4 | 0.8763 |
| Salmeterol | No Response |  |  |  |  | No Response |  |  |  |
| Xamoterol | No Response |  |  |  |  | No Response |  |  |  |
|  | G12 | | | |  | G12-p115 | | | |
|  | pEC50 | CI-95 | n | Comparison ISO |  | pEC50 | CI-95 | n | Comparison ISO |
| Isoproterenol | 7.83 ± 0.14 | 7.54 - 8.11 | 9 |  |  | 8.21 ± 0.07 | 8.08 - 8.35 | 3 |  |
| Epinephrine | 6.64 ± 0.23 | 6 .17 - 7.11 | 4 | 0.1126 |  | 7.00 ± 0.11 | 6.78 - 7.23 | 3 | 0.0004 |
| Norepinephrine | 6.28 ± 0.24 | 5.78 - 6.77 | 3 | 0.0501 |  | 7.45 ± 0.05 | 7.35 - 7.56 | 3 | 0.0069 |
| Isoetharine | 6.63 ± 0.72 | 5.15 - 8.10 | 4 | 0.1085 |  | No Response |  |  |  |
| Indacaterol | 6.54 ± 0.56 | 5.40 - 7.67 | 5 | 0.0517 |  | 7.19 ± 0.21 | 6.76 - 7.61 | 3 | 0.0011 |
| Salmeterol | No Response |  |  |  |  | No Response |  |  |  |
| Xamoterol | No Response |  |  |  |  | No Response |  |  |  |
|  | Ca^2+^ (Obelin) | | | |  | βarrestin2 | | | |
|  | pEC50 | CI-95 | n | Comparison ISO |  | pEC50 | CI-95 | n | Comparison ISO |
| Isoproterenol | 7.68 ± 0.06 | 7.56 - 7.80 | 3 |  |  | 7.67 ± 0.04 | 7.59 - 7.76 | 4 |  |
| Epinephrine | 7.20 ± 0.11 | 6.98 - 7.43 | 3 | 0.8155 |  | 6.16 ± 0.08 | 6.00 - 6.33 | 4 | 0.7187 |
| Norepinephrine | 7.17 ± 0.06 | 7.05 - 7.29 | 3 | 0.7802 |  | 6.68 ± 0.06 | 6.55 - 6.80 | 4 | 0.9095 |
| Isoetharine | No Response |  |  |  |  | No Response |  |  |  |
| Indacaterol | 5.80 ± 0.25 | 5.29 - 6.31 | 3 | 0.0245 |  | 6.09 ± 1.06 | 3.97 - 8.22 | 4 | 0.6876 |
| Salmeterol | No Response |  |  |  |  | No Response |  |  |  |
| Xamoterol | No Response |  |  |  |  | No Response |  |  |  |

HA-β_1_AR-expressing HEK 293 cells were stimulated with increasing concentrations of the indicated ligands and responses were measured for 8 different biosensors. Data were fit with logistic 3 or 4 parameter equations to generate pEC_50_ values, expressed as mean ± SEM and corresponding confidence intervals (CI-95). pEC_50_ values for each ligand were compared to isoproterenol pEC_50_ in corresponding biosensors using one-way ANOVA followed by Dunnett’s test and p values indicated in the “Comparison ISO” column. “n” corresponds to the number of independent experiments for indicated ligand and biosensor. Data were derived from experiments presented in **Fig. 2e** (Gα_s_), **Fig. 2f** (cAMP (EPAC)), **Fig. 3c** (Gα_i2_), **Fig. 4d** (Gα_z_), **Fig. 5e** (Gα_12_), **Fig. 5f** (Gα_12_-p115), **Fig. 7c** (Ca^2+^ (Obelin)) and **Fig. 8c** (β-arrestin2).

**Supplementary Table S3:** *Transduction coefficients (log(τ/K_A_)) for ligand effects on the β_1_AR.*

|  | **Log(τ/K_A_)** | | | | | | | |
| --- | --- | --- | --- | --- | --- | --- | --- | --- |
|  | **Gαs** | **EPAC** | **Gαi2** | **Gαz** | **Gα12** | **Gα12-p115** | **Obelin** | **βarrestin2** |
| Isoproterenol | 7.99 ± 0.13 | 9.84 ± 0.09 | 7.03 ± 0.06 | 7.41 ± 0.05 | 7.67 ± 0.20 | 8.28 ± 0.05 | 7.69 ± 0.07 | 7.70 ± 0.05 |
| Epinephrine | 7.13 ± 0.18 | 8.36 ± 0.10 | 5.72 ± 0.06 | 6.10 ± 0.04 | 6.18 ± 0.27 | 6.83 ± 0.05 | 7.24 ± 0.07 | 6.12 ± 0.05 |
| Norepinephrine | 6.89 ± 0.23 | 8.96 ± 0.12 | 6.21 ± 0.06 | 6.60 ± 0.04 | 6.45 ± 0.26 | 7.45 ± 0.06 | 7.13 ± 0.07 | 6.64 ± 0.05 |
| Isoetharine | 5.62 ± 0.18 | 6.98± 0.10 | No Response | No Response | 4.31 ± 0.86 | No Response | No Response | No Response |
| Indacaterol | 6.72 ± 0.18 | 8.61 ± 0.10 | No Response | 6.62 ± 0.24 | 4.96 ± 0.55 | 6.44 ± 0.24 | No Response | 5.95 ± 0.18 |
| Salmeterol | 5.29 ± 0.31 | 6.50 ± 0.11 | No Response | No Response | No Response | No Response | No Response | No Response |
| Xamoterol | 6.61 ± 0.55 | 8.46 ± 0.19 | No Response | No Response | No Response | No Response | No Response | No Response |

Dose-response curves for indicated ligands were also analyzed with the operational model to yield log(τ/K_A_) ratios^16,17^, expressed as mean ± SEM.

**Supplementary Table S4. Expression of Gα subunits in HEK 293PL cells.** Expression of each Gα is presented as the average reads per kilobase per million reads (RPKM) of two biological replicates.

| **Gene** | **RPKM** |
| --- | --- |
| GNA11 | 34.0 |
| GNA12 | 18.6 |
| GNA13 | 24.9 |
| GNA14 | 0.0 |
| GNA15 | 0.0 |
| GNAI1 | 6.6 |
| GNAI2 | 41.3 |
| GNAI3 | 5.4 |
| GNAL | 9.3 |
| GNAO1 | 0.3 |
| GNAQ | 11.9 |
| GNAS | 244.8 |
| GNAT1 | 0.0 |
| GNAT2 | 0.2 |
| GNAT3 | 0.0 |
| GNAZ | 10.8 |
